# *Oleaeuropaea* leaf exosome-like nanovesicles encapsulated in a hyaluronic acid / tannic acid hydrogel dressing with dual “defense-repair” effects for treating skin photoaging

**DOI:** 10.1016/j.mtbio.2024.101103

**Published:** 2024-05-31

**Authors:** Zhenzhen Wang, Jumao Yuan, Yan Xu, Nuo Shi, Lin Lin, Ruirui Wang, Rong Dai, Lin Xu, Ning Hao, Qianyi Li

**Affiliations:** a Peterson's Lab, Shanghai, PR China; b International Laboratory in Cancer, Aging and Hematology, Shanghai Jiao Tong University, School of Medicine/Ruijin Hospital/CNRS/Inserm/Côte d'Azur University, Shanghai, PR China; c Pôle Sino-Français de Recherches en Sciences du Vivant et G'enomique, Shanghai, PR China; d Department of Emergency, Ruijin Hospital, Shanghai Jiaotong University School of Medicine, Shanghai, PR China; e Institute of Symbolcell Biotechology, Nanjing, Jiangsu, PR China; f Baudry Biotech. Co., Ltd, Nanjing, Jiangsu, PR China; g College of Biotechnology and Pharmaceutical Engineering, Nanjing Tech University, Nanjing, Jiangsu, PR China

**Keywords:** Photoaging, UV shielding, Exosome-like nanovesicles, Extracellular vesicles, Hyaluronic acid hydrogel, Skin regeneration

## Abstract

Photoaging, primarily caused by ultraviolet (UV) light, is the major factor in extrinsic skin aging. Existing anti-photoaging strategies mainly focus on early sun protection or repairing damaged skin, lacking a comprehensive treatment strategy. Therefore, this study developed a dressing that actively shields against UV radiation and repairs photoaged skin, offering double protection. This study utilized exosome-like nanovesicles derived from *Olea europaea* leaves (OLELNVs), enhancing them into a potent core biomaterial with high-dose effects and skin-friendly, non-cytotoxic inhibition of cell aging. These nanovesicles were incorporated into a cross-linked hyaluronic acid (HA) and tannic acid (TA) hydrogel with strong UV-absorbing properties, creating the OLELNVs@HA/TA hydrogel system. *In vitro* and *in vivo* experiments demonstrated that OLELNVs@HA/TA hydrogel can effectively reduce UV-induced skin damage and promote skin repair and regeneration. Additionally, RNA-seq and clustering analysis of miR168a-5p predicted targets revealed significant down-regulation of the NF-κB signaling pathway, mediating inflammatory aging responses. Overall, the OLELNVs@HA/TA hydrogel represents a novel dual-strategy approach for clinical application in combating photoaging.

## Graphical abstract

**Figure:**
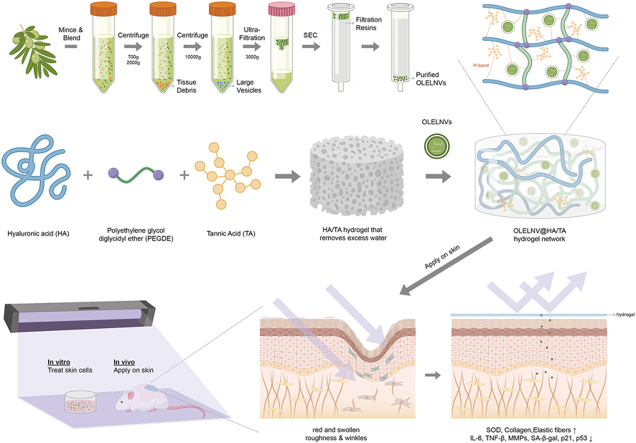

## 1 Introduction

Photoaging is the primary environmental cause of skin aging in humans, predominantly due to exposure to ultraviolet (UV) radiation from the sun [1]. UV radiation accelerates skin cell aging through direct effects like DNA damage and indirect effects such as oxidative stress. Senescent cells, following skin damage, release cytokines, chemokines, growth factors, proteases, and extracellular vesicles, collectively known as the senescence-associated secretory phenotype (SASP) [2,3]. This results in a pro-inflammatory and pro-aging environment that expedites the aging of adjacent normal cells, thereby perpetuating a vicious cycle of skin aging. Consequently, the structural and functional integrity of the skin deteriorates, leading to conditions such as epidermal hyperkeratosis with dysplasia, wrinkles, loss of elasticity, dryness, sagging and rough texture. This deterioration may also precipitate the development of precancerous and cancerous lesions [4,5]. Existing strategies against UV photoaging, which primarily focus on blocking UV radiation or repairing damaged skin, are inadequate. Integrating protective and reparative functions in new material development appears essential for effectively combating the impacts of UV radiation. Therefore, a novel dressing has been developed that offers both passive protection and active repair, countering the effects of photoaging on skin cells.

*Olea europaea* leaf extract (OLEX) is extensively employed in various skincare and topical dressing formulations due to its significant antioxidant, anti-inflammatory and anti-aging properties. Studies have demonstrated that OLEX enhances skin cell viability by inhibiting fibroblast apoptosis and delivers anti-inflammatory and anti-aging benefits through the AP-1 and NF-κB signaling pathways following skin injury [6,7]. Moreover, distinct from typical skin aging, photoaging precipitates a swift accumulation of senescent cells and considerable thickening of the stratum corneum, significantly impeding the absorption of water-soluble OLEX [8]. In efforts to amplify anti-aging effects, increasing the concentration of bioactive components frequently leads to heightened cytotoxicity, thereby restricting the extract's utility in dermatological treatments. Based on this, the use of exosome-like nanovesicles derived from *O. europaea* leaves has been investigated to enable more precise dosage control and minimize adverse effects.

In recent years, the study of plant exosome-like nanovesicles (PELNVs) — small vesicles released by plant cells containing bioactive molecules such as proteins, nucleic acids and lipids — has garnered widespread interest in the scientific community [[9], [10], [11]]. PELNVs offer potential for drug delivery in medicine and provide an innovative, biocompatible approach to natural skincare [12,13]. With the successful extraction of *O. europaea* leaf exosome-like nanovesicles (OLELNVs), our findings reveal their significant anti-photoaging properties, enhanced skin and cellular absorption, and excellent biocompatibility and safety. Consequently, OLELNVs have been employed as a core material to combat photoaging and mitigate the accumulation of senescent cells.

Hydrogel is widely regarded as a biomaterial with enormous potential, particularly due to its outstanding hydration ability and biomimetic properties. These qualities have shown great potential in various fields such as medicine, drug delivery, tissue engineering and biosensing. The porous 3D network structure of the hydrogel serves as a basis for the uptake of exosomes. On the one hand, hydrogels loaded with exosomes can effectively restrict their Brownian motion thus ensuring exosome retention [14]. On the other hand, the topical application of exosomes encapsulated in hydrogels forms a film on the skin surface, enhancing their retention time under harsh conditions and facilitating sustained release on the skin [15]. Furthermore, a hydrogel network has been engineered through the covalent and physical cross-linking of hyaluronic acid (HA), polyethylene glycol diglycidyl ether (PEGDE), and tannic acid (TA). In this network, HA enhances moisture content, slows skin aging, and promotes the repair of skin damage. Notably, the addition of a specific proportion of TA to HA endows the hydrogel with excellent UV absorption properties, without altering its physical characteristics. This efficacy is primarily attributed to the multiple aromatic rings and hydroxyl functional groups in TA molecules, which exhibit strong absorption peaks in the UV region. Consequently, the HA/TA hydrogel network acts as a protective agent for encapsulated exosomes and a shield against harmful UV radiation as well.

To provide dual protection against photo-aging, the OLELNVs@HA/TA hydrogel dressing has been developed, which not only shields the skin from UV radiation but also counteracts the accumulation of senescent cells resulting from UV exposure. *In vitro* studies have demonstrated that the OLELNVs@HA/TA hydrogel effectively guards against UV damage, offering comprehensive protection against UV radiation cells. *In vivo* experiments have shown that topical application of the OLELNVs@HA/TA hydrogel in a mouse skin photoaging model effectively reduces oxidative stress and interrupts the detrimental cycle of aging factors in cells. This treatment enhances the production of skin collagen and elastin, thus facilitating skin repair and regeneration. Furthermore, bioinformatics analyses, including clustering analysis and pathway prediction of predicted human target genes, reveal significant regulatory effects on the NF-κB signaling pathway, which is crucial in mediating inflammatory aging responses. In summary, the OLELNVs@HA/TA hydrogel design introduces a novel approach for the clinical application of dual “defense-repair” strategies against photoaging.

## 2 Materials and methods

### 2.1 Materials

*O. europaea* leaves were sourced from local olive farms in Mianyang, Sichuan, China. The *O. europaea* extract (EUROL®BT) was from Hallstar, USA. DAPI was obtained from Thermo Fisher, USA. Hyaluronic acid (HA) was purchased from Freda Bio, China. Polyethylene glycol diglycidyl ether (PEGDE), ABTS, potassium persulfate (K_2_S_2_O_8_) and sodium carbonate (Na_2_CO_3_) were obtained from Aladdin, China. PKH67, tannic acid, gallic acid were provided from Sigma Aldrich, USA. The Cell Counting Kit-8 (CCK-8) was obtained from Beyotime Biotech, China. Folin-Ciocalteu reagent was purchased from Merck, Germany.

### 2.2 Cells

The human immortal keratinocyte cell line (HaCaT) was purchased from Keygen Biotech (Nanjing, China) and the human dermal fibroblast cells (HDF-α) were purchased from Jennio Biotech (Guangzhou, China). Both cell lines were cultured in DMEM medium (Gibco, USA) with 10 % heat-inactivated fetal bovine serum (Hyclone, New Zealand) at 37 °C in a humidified atmosphere with 5 % CO₂.

### 2.3 Animals

Specific pathogen Free (SPF) male Institute of Cancer Research (ICR) mice (aged 6–8 weeks) were provided by Clinbridge Biotech (Nanjing, China) and acclimatized for 7 days prior to the experiments. All animal experiments were conducted in accordance with the ethical guidelines approved by the Institutional Animal Care and Use Committee, Clinbridge Biotech (Ethics approval number: AP-C230806).

### 2.4 OLELNVs separation

*O. europaea* leaves were washed with distilled water to remove dust and soil. The juice was extracted using a blender and then centrifuged at 700 g for 10 min, 2000 g for 20 min, and 10,000 g for 30 min to remove large plant fragments. Amicon Ultra-15 (Millipore, USA) centrifugal filters were used to concentrate the juice followed by separation of the exosome-like nanovesicles using a size-exclusion chromatography (Exosupur® columns, Echobiotech, China). The isolated OLELNVs were resuspended in PBS, sterilized by filtration through a 0.22 μm filter (Merck Millipore, USA) and stored at −80 °C.

### 2.5 Characterization of OLELNVs

The particle size, concentration and zeta potential of OLELNVs were measured using Nanoparticle Tracking Analysis (Particle Metrix, Germany) and their morphology was observed using Transmission Electron Microscopy (Hitachi, Japan).

### 2.6 Fluorescence microscopy of OLELNVs uptake into cells

OLELNVs were fluorescently labeled with the green dye PKH67 and incubated with HaCaT cells according to the manufacturer's recommended protocol. PKH67 was diluted and mixed with diluent C. OLELNVs were incubated with the PKH67 dye for 15 min at 25 °C, and bovine serum albumin (FBS) was added to stop the staining. Ultrafiltration with Amicon Ultra-4 was used to remove the free PKH67 dye. HaCaT cells were incubated with PKH67-labeled OLELNVs for 24 h. The cells were then fixed with 4 % paraformaldehyde and stained with the fluorescent dye DAPI for 10 min. After several washes, the intracellular uptake of OLELNVs was analyzed using an inverted fluorescence microscope (Leica, Germany).

### 2.7 OLEX preparation

EUROL®BT consists of 8 % raw material, 60 % fructose, and 32 % water. EUROL®BT was diluted with PBS to obtain OLEX concentrations ranging from 0.1 to 1 mg/ml, according to calculations.

### 2.8 Cell viability assay

The Cell Counting Kit 8 (CCK8) assay was used to assess cell proliferation according to the manufacturer's recommended protocol. HaCaT cells (8000 cells/well) were seeded in 96-well plates (Corning, USA) and incubated until the cells adhered. For the UV-induced model assay protocol, cells were treated with UVB (300 mJ/cm^2^, Philip, Netherlands) with an emission spectrum of 311 nm and then treated with either OLELNVs/OLEX or PBS and co-incubated for 24 h. To each well, 10 μL of CCK8 solution was added and incubated for an additional hour. Optical density (OD) was measured at 450 nm using a multifunctional microplate reader (Molecular Devices, USA).

### 2.9 Cell scratch assay

HaCaT cells were used for the cell scratch test. HaCaT cells (1 × 10^6^ cells/well) were seeded in 6-well plates and cultured for 1–2 days until fully confluent. Confluent monolayers of HaCaT cells were scratched with pipette tips. Detached cells and debris were removed by washing with PBS. Cells were then treated with or without OLELNVs/OLEX (0.1 mg/mL & 1 mg/ml) in DMEM containing 5 % fetal bovine serum. The scratch area was photographed at 0 h, 12 h and 24 h after scratch formation and quantified using ImageJ software. The percentage of wound closure at each time point was calculated based on the initial scratch size at 0 h. Images were obtained using a DMi1 inverted phase-contrast microscope (Leica, Germany).

### 2.10 Transwell assay

HaCaT cells were initially treated with OLELNVs for 24 h. Following this treatment, the cells were suspended in serum-free medium at a density of 5 × 10^5^ cells/mL. Next, 100 μL of this cell suspension was placed in the inverts of a 24-well Transwell plate with an 8 μm pore size (Corning, NY, USA). The lower chamber was filled with a culture medium containing 5 % FBS. After incubating for 48 h, cells that had not migrated were removed from the inverts using cotton swabs, and the chamber was fixed with 4 % paraformaldehyde. Cells that migrated through the membrane were stained with crystal violet for 15 min. Images of these cells were captured using a DMi1 inverted phase-contrast microscope (Leica, Germany).

### 2.11 ABTS radical scavenging assay

The antioxidant activity of OLELNVs/OLEX was evaluated using the ABTS radical scavenging assay. A 7.4 mM ABTS solution was mixed with a 2.6 mM potassium persulfate solution in a 1:1 ratio and stored in the dark for 12 h. This mixture was then diluted with PBS to obtain the ABTS + working solution. For the assay, 0.8 mL of the ABTS + solution was mixed with 0.2 mL of the sample or distilled water, shaken, and left for 6 min. Absorbance was measured at 734 nm using a UV–Vis spectrophotometer (Agilent, USA). Antioxidant capacity was calculated as: Free radical scavenging (%) = *(A₀* *- A)* */* *A₀* *× 100 %*, where *A₀* is the control absorbance and *A* is the sample absorbance.

### 2.12 Total phenolic compounds (TPC) assay

TPC in OLELNVs was measured using the Folin-Ciocalteu method. We mixed 0.5 mL of OLELNVs in PBS with 0.5 mL of 0.25 M Folin-Ciocalteu reagent and 1 mL of 15 % sodium carbonate. After 30 min of incubation and 3 min of centrifugation at 3500 RPM, absorbance was measured at 760 nm using a UV–Vis spectrophotometer (Agilent, USA). TPC values were reported as milligrams of gallic acid equivalents (GAE) per liter of OLELNVs, using a standard curve.

### 2.13 High-performance liquid chromatography-mass spectrometry (HPLC/MS)

The analysis of samples was conducted using an HPLC-MS method (Shimadzu LCMS-8050) employing positive ionization mode with multiple reaction monitoring (MRM) for hydroxytyrosol and oleuropein. Separation was equipped with a photodiode array detector and an Endeavorsil column (Leapsil 2.7 μm C18 100*2.1 mm). A gradient method employing mobile phase A (0.1 % formic acid in water) and mobile phase B (acetonitrile) was used to separate the individual OLELNVs components. Data acquisition and quantification were performed using SHIMADZU Labsolutions.

### 2.14 Antioxidant enzymes assay

The contents of Total Superoxide Dismutase (T-SOD) encapsulated in OLELNVs were determined by assay kits (NJJCbio, China).

### 2.15 Cell function assays

The Sa-beta-gal staining kit (Beyotime Biotech, China) was used to detect SA-β-gal activity according to the manufacturer's recommended protocol. Cu/Zn-SOD and Mn-SOD assay kit with WST-8 (Beyotime Biotech, China) was used to detect SOD activity. The Reactive oxygen species (ROS) assay kit (Beyotime Biotech, China) was used for the detection of ROS. The intracellular concentration of the inflammatory cytokine interleukin 6 (IL-6) was measured using ELISA kits (Multisciences, China).

### 2.16 Western blot analysis

Proteins were extracted from tissues and cells, separated by 10 % SDS-PAGE, and transferred to preactivated polyvinylidene fluoride (PVDF) membranes (Millipore, Bedford). The protein-containing membranes were sealed with 5 % skim milk powder (Solarbio, China) and incubated overnight at 4 °C with the specific primary antibodies COL-I (Abcam, 1:5000 dilution), MMP1 (Abcam, 1:5000 dilution), MMP3 (Abcam, 1:5000 dilution), GAPDH (Abcam, 1:5000 dilution). After washing with TBST, membranes were incubated with HRP-conjugated secondary antibodies for 1 h at 37 °C. Color development was performed with ECL reagent (Millipore, MA, USA). Images were captured with the Bio-Rad Gel Imaging System. GAPDH was used as a loading control.

### 2.17 Transcriptome sequencing

Total RNA was extracted from UVB-induced HaCaT and treated with OLELNVs. Gene expression profile changes were analyzed by transcriptome sequencing. mRNA with polyA structure was captured using magnetic beads with Oligo (dT). The first strand of cDNA was synthesized using fragmented mRNA as a template in the M-MuLV reverse transcriptase system, followed by second strand synthesis. Double-stranded cDNA was purified, end-repaired, A-tailed, and ligated with sequencing adapters. Approximately 200 bp cDNA was selected, amplified by PCR, and purified to obtain the sequencing library. Illumina PE150 sequencing was performed, followed by bioinformatic analysis.

### 2.18 miRNA sequencing

Total RNA was extracted from OL juice and OLELNVs, and quality was checked using the Qsep100 automated nucleic acid protein analysis system (Bioptic, USA). The miRNA sequencing library was prepared and sequenced using the PE150 protocol. Fastqc was used for quality evaluation, and fastp for N-base excision, q20 filtering, and adapter removal. Clean sequences were aligned to the Rfam library using Bowtie to remove rRNA, tRNA, and other ncRNA, followed by alignment to the genome. Known miRNAs in OLELNVs were identified by aligning sequences to official databases. Target genes of miRNA candidates were predicted using TargetScan and miRanda algorithms. The function of the target gene was determined using Kyoto Encyclopedia of Genes and Genomes (KEGG) databases.

### 2.19 Preparation of hydrogels

PEGDE (0.6g, Mn 2000) was fully dissolved in 100 ml of 0.1 g/L NaOH. Then, HA (6g, Mw = 1.3–1.5 million kDA) was added to cross-link and maintained at 25 °C for 24 h. The HA hydrogels were washed with 0.9 % NaCl to remove any residues and adjusted pH = 7.4 ± 0.5 with dilute HCl. Afterwards, the HA hydrogels were immersed in TA solution with different concentrations (0.01 %, 0.02 %, 0.03 %, 0.04 %, 0.05 % and 0.5 %) for 24 h. The TA-treated HA hydrogels were washed with phosphate-buffered saline (PBS). For the hydrogels loaded with OLELNVs, OLELNVs were mixed with HA/TA hydrogels subsequently.

### 2.20 Hydrogels characterization

The hydrogels' structural changes were analyzed using attenuated total reflectance infrared spectroscopy (ATR-IR) (Agilent Cary 630 FTIR). Spectra were recorded in the 650–4000 cm⁻^1^ range. Morphology was observed using scanning electron microscopy (SEM) (Zeiss Sigma 300, Germany). Viscoelasticity was measured with a HAAKE™ MARS™ 60 Rheometer (Germany). Frequency sweeps were conducted at room temperature with constant strain (10 % in the linear viscoelastic range) over 0.1–100 rad/s. The sample was equilibrated for 3 min before testing, with 34 data points collected. Strain sweeps were performed with angular frequency at 10 rad/s, and shear strain ranged from 0.01 % to 1000 %. Parameters for frequency sweeps: Gap Size = 3000 μm, Frequency = 0.1–20 rad/s, Strain = 2 %, Temperature = 37.0 °C. Parameters for strain sweeps: Gap Size = 3000 μm, Frequency = 2.0 rad/s, Strain = 0.1–1000 %, Temperature = 37.0 °C.

### 2.21 Release kinetics of hydrogels encapsulated OLELNVs

To detect OLELNVs released from HA/TA hydrogels, OLELNVs were mixed with HA/TA and then placed in the Transwell inserts in a 24-well plate (Corning, NY, USA) and added 100 μL of PBS to the lower chamber. Samples were collected from the lower chamber after 12 h, 24 h, 48 h, and 72 h. Protein concentration in the lower chamber was quantified using the Bradford assay (Bio-Rad, USA) to calculate the percentage of OLELNVs released.

### 2.22 In vitro UV resistance evaluation

The absorbance of samples was measured from 200 to 800 nm using a UV–Vis spectrophotometer (Agilent Cary 60 UV–Vis, USA), with deionized water as the baseline. HA/TA solutions with different TA concentrations (0 %, 0.01 %, 0.02 %, 0.03 %, 0.04 %, and 0.05 %) with or without OLELNVs were tested. Dermal fibroblasts (HDF-α) were seeded in 96-well plates at 8000 cells/well and incubated in a humidified CO_2_ incubator at 37 °C for 24 h. The cell culture medium was then replaced with PBS buffer. The cells were divided into 9 groups: control, model, OLELNVs, HA, HA/TA (TA: 0.01 %, 0.05 %), OLELNVs@HA, and OLELNVs@HA/TA (TA: 0.01 %, 0.05 %). In the UVB blocking model, samples were placed 1 cm above the cell culture plate on quartz plates, and cells were treated with UVB (300 mJ/cm^2^, Philips, Netherlands, 311 nm). In the UVB damage repair model, cells were first treated with UVB, then samples were placed in Transwell inserts and incubated in DMEM complete medium for 48 h. The blank control group received no UV radiation and the model group received only UV radiation. After 10 μL of CCK8 solution was added to each well and incubated for 1 h, optical density (OD) was measured at 450 nm using a multifunctional microplate reader (Molecular Devices, USA).

Additionally, HDF-α cells (3 × 10^5^ cells/well) were seeded in 24-well plates and divided into 8 groups: control, model, OLELNVs, OLEX, HA, HA/TA (0.05 % TA), OLELNVs@HA, and OLELNVs@HA/TA (0.05 % TA). The same UVB blocking model and UVB damage repair model were used. Cells were then digested with trypsin (Gibco, USA), stained with the LIVE/DEAD® Cell Viability/Cytotoxicity Kit (Thermo Fisher, USA), and observed using an inverted fluorescence microscope (Leica, Germany). ImageJ software was used to count and analyze live and dead cells.

### 2.23 Skin penetration capacity evaluation

Hair was removed from the back of mice and the skin tissues were obtained the next day for immediate use. Dil dye, Dil-labeled OLELNVs, dansyl chloride (DNS) dye and DNS-labeled OLEX were applied to the skin tissues at room temperature for 8 h. The skin tissues were then washed and sliced into sections and frozen in paraffin using standardized protocols. Whole skin samples were collected, photographed and scanned with a 3D white light fluorescence scanner (Pannoramic MIDI II, Hungary) for subsequent digital image analysis.

### 2.24 Skin integration capacity evaluation

For the skin adhesion study, hair was removed from the back of ICR mice, OLELNVs were labeled with Dil (OLELNVs-Dil), and a portion of the labeled OLELNVs was loaded into HA/TA hydrogel (OLELNVs@HA/TA-Dil). Both OLELNVs-Dil and OLELNVs@HA/TA-Dil were applied to the mice's back skin. Samples remaining on the skin were imaged and quantified using the IVIS®Lumina III (PerkinElmer, USA). To evaluate sweat resistance, both groups were sprayed with artificial sweat (0.5 % NaCl, 0.1 % urea, 0.1 % lactic acid, pH adjusted to 6.6 with NH_4_OH) 30 min after application of the samples, followed by IVIS imaging and analysis.

### 2.25 In vivo anti-photoaging evaluation

After back hair removal, ICR mice were randomly divided into 6 groups (n = 10): Control, Model, OLELNVs (1 mg/ml), HA/TA (TA: 0.5 %), OLELNVs@HA/TA (OLELNVs: 1 mg/ml; TA: 0.5 %), and OLEX (1 mg/ml). The control group was kept normal, while the treatment groups were irradiated with UVB every other day for 4 weeks. The HA/TA group received treatment before each irradiation (for sun blocking), the OLELNVs and OLEX groups received treatment after each irradiation (for repairing), and the OLELNVs@HA/TA group received treatment both before and after each irradiation (for dual effects). The model group was treated with saline as placebo. UV irradiation intensity started at 1 MED (500 mJ/cm^2^) for the first 2 weeks, increased to 2 MED in the third week, and 3 MED in the fourth week. Skin changes were photographed weekly. After 4 weeks, dorsal skin was excised, with fresh tissue portions soaked in PBS for biochemical detection of SOD and IL-6, and other portions fixed in 10 % formalin and embedded in paraffin for Hematoxylin-Eosin (H&E) staining, Fontana-Masson staining, immunohistochemical and immunofluorescence staining.

### 2.26 Immunohistochemical staining

Paraffin-embedded skin slices were incubated with xylene and graded ethanol, washed 3 times, then transferred to a target recovery solution containing sodium citrate and boiled for repair. Slices were incubated in 3 % hydrogen peroxide solution for 15 min at room temperature and protected from light. The samples were then placed in goat serum blocking solution for 30 min. Primary antibodies (ab270993 and ab52915, Abcam) were added and incubated overnight at 4 °C. After washing with PBS, tissue slices were coated with a secondary antibody (HRP-labeled, Thermofisher) corresponding to the primary antibody and incubated for 50 min at room temperature. The DAB peroxidase (HRP) substrate kit (Thermofisher, USA) was used for color development, and hematoxylin, differentiation solution and cyanogen solution were added sequentially for nuclear counterstaining. Finally, the slides were dehydrated in absolute ethanol and xylene, sealed and viewed under the microscope.

### 2.27 Immunofluorescence staining

Deparaffinization of paraffin slices to water and antigen retrieval follows the above-mentioned method. Primary antibodies (TUNEL, p53, p21, TGF-β) were added and incubated overnight at 4 °C. Slices were then incubated with secondary antibody at room temperature for 1 h, shielded from light, and examined under a Leica DMI6000B fluorescence microscope (Germany).

### 2.28 Statistical analysis

All experiments, except miRNA-seq, were repeated independently at least 3 times, with data expressed as mean ± SD. Raw miRNA analysis values were normalized using the tag count per million aligned miRNAs (TPM) method. Statistical analysis and graphs were conducted using GraphPad Prism 9 (GraphPad Software Inc., USA). Statistical comparisons were performed using Student's t-test (two groups) and one-way ANOVA (three or more groups). P-values of 0.05(*),0.01 (**), 0.001 (***) were statistically significant.

## 3 Results

OLEX is a widely utilized skincare ingredient in the formulations of products and topical dressings from leading brands. However, it exhibits several limitations, including low transdermal efficiency due to its water-soluble nature, which impedes penetration through the stratum corneum, and cytotoxicity at high concentrations despite its efficacy. Consequently, this study explores whether exosome-like nanovesicles derived from the same plant can address these challenges.

### 3.1 Preparation and characterization of OLELNVs

**Fig. 1:**
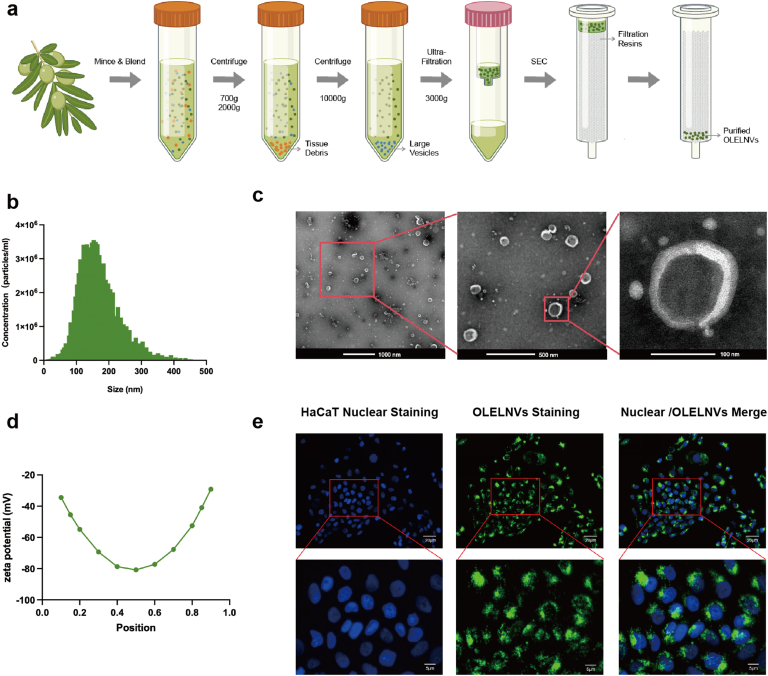
Preparation and characterization of OLELNVs. (a) Flow chart illustrating the isolation of OLELNVs by ultrafiltration and size exclusion chromatography. (b) The average size of OLELNVs characterized by Zetaview analysis. (c) Representative TEM image of purified OLELNVs. Scale bars = 100 nm, 500 nm & 1000 nm. (d) Zeta potential of OLELNVs. (e) Tracing the uptake of OLELNVs by HaCaT cells. OLELNVs were labeled with PKH-67 (green) and HaCaT cell nuclei were labeled with DAPI (blue). Scale bar = 20 μm, 5 μm. (For interpretation of the references to colour in this figure legend, the reader is referred to the Web version of this article.)

To investigate the biological and pharmacological activities of OLELNVs, their isolation from *O. europaea* leaves in high yield and high purity is crucial. A combination of ultrafiltration and size exclusion chromatography was utilized to isolate the nanovesicles. (Fig. 1a). To further characterize the OLELNVs, nanoparticle tracking analysis (NTA) and transmission electron microscopy (TEM) were used to assess their size, zeta potential and morphology. The size distribution of OLELNVs peaked at approximately 140 nm in diameter (Fig. 1b), and the nanovesicles exhibited a circular shape with distinct exosome-like lipid bilayer membrane structure features (Fig. 1c). The purity of OLELNVs was determined to be 1.8 × 10^12^ particles/mg, surpassing the industry's purity standard of 1.0 × 10^11^ particles/mg [16,17]. The average zeta potential was −40.71 ± 0.28 mV (Fig. 1d), indicating a stable, negatively charged sample.

Furthermore, the cellular uptake of OLELNVs was evaluated, which is crucial as these nanovesicles interact with cells to transfer bioactive molecules, thus influencing cellular functions. To assess penetration and localization, OLELNVs labeled with PKH67 were incubated in the HaCaT cell culture medium. Fluorescence microscopy revealed that OLELNVs were present in the cytoplasm of the cells (Fig. 1e), demonstrating that OLELNVs can penetrate human cells and transport molecular cargo, thereby modulating the biological activities of the target cells.

### 3.2 Enhanced safety and efficacy of OLELNVs compared to OLEX

To ascertain if OLELNVs mirror the effects of traditional *O. europaea* leaf extracts while leveraging the advantages of PELNVs, a series of controlled experiments comparing OLELNVs to OLEX were performed.

**Fig. 2:**
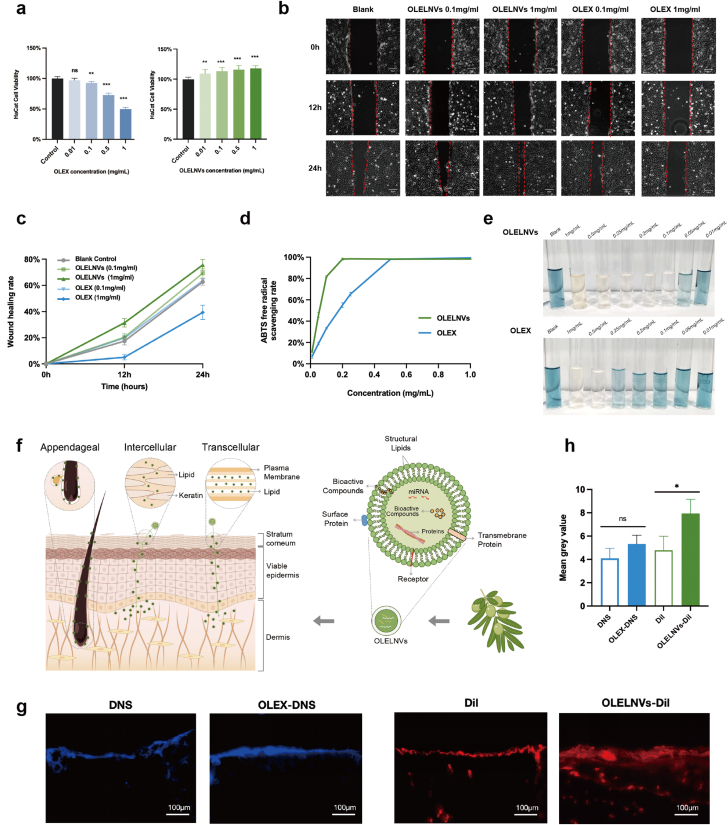
Enhanced safety and efficacy of OLELNVs compared to OLEX. (a) The effect of OLELNVs and OLEX on HaCaT cell proliferation by CCK8 assay. (b, c) The effect of OLELNVs and OLEX on the wound healing of HaCaT cells, and the wound healing rates of each group after 12 and 24 h. Scale bar = 50 μm, n = 3. (d, e) The activity of OLELNVs and OLEX in scavenging ABTS free radicals, n = 3. (f) Schematic representation of the penetration of PELNVs through different pathways in the skin. (g, h) The penetration effect of OLELNVs and OLEX in skin slices with topical application; OLEX labeled with DNS (blue) and OLELNVs with Dil (red). Scale bar = 100 μm, n = 3. Data are expressed as mean ± SD (*P＜0.05, NS: not significant). (For interpretation of the references to colour in this figure legend, the reader is referred to the Web version of this article.)

PELNVs, as natural nanovesicles, have been reported to exhibit low cytotoxicity due to their cellular derivation and composition [18]. To validate this, CCK8 assays were employed to assess the cytotoxicity of OLELNVs and OLEX on HaCaT cell proliferation at various concentrations. The results indicated that OLEX, like most organic solvent-derived plant extracts, displayed dose-dependent toxicity; cell viability was 91 % at 0.1 mg/ml, decreased to 73 % at 0.5 mg/ml, and reduced by half at 1 mg/ml. In contrast, OLELNVs maintained the same biocompatibility as PELNVs, showing no toxicity to HaCaT cells at any tested concentration. Moreover, HaCaT cell viability improved with increasing concentrations of OLELNVs, suggesting a potential cell proliferation-promoting effect (Fig. 2a).

To further validate the effect of OLELNVs and OLEX on cell migration and proliferation, scratch-wound experiments were conducted. HaCaT cells were scratched and subsequently incubated with low (0.1 mg/ml) and high (1 mg/ml) doses of OLELNVs and OLEX, respectively. Compared to the empty control group, the OLELNVs-treated cells exhibited faster scratch closure in a dose-dependent manner. Conversely, the OLEX group demonstrated a modest increase in scratch closure rate at the low dose compared to the control, but this rate decreased significantly with higher concentrations (Fig. 2b). After 24 h, the closure rate in the high-dose OLELNVs group reached 76 ± 2 %, whereas it was only 40 ± 3 % in the high-dose OLEX group (Fig. 2c). Further assessments using Transwell assays at a safe and low concentration (0.1 mg/mL) consistently demonstrated that, unlike OLEX, OLELNVs significantly enhance the migration of HaCaT cells (Fig. S3). These tests reiterate that OLELNVs more effectively promote cell growth and migration compared to OLEX.

OLEX is renowned for its high content of bioactive compounds, including potent antioxidant phenolic compounds such as oleuropein and hydroxytyrosol [19]. To determine if OLELNVs share these antioxidant properties, the ABTS radical scavenging method, a popular technique for evaluating the free radical scavenging capacity of samples, was utilized [20]. This method measures the reduction in absorbance at 734 nm as antioxidants neutralize ABTS radicals. Both OLEX and OLELNVs displayed dose-dependent absorbance attenuation across concentrations from 0.01 mg/ml to 1 mg/ml (Fig. 2e). Notably, at 0.1 mg/ml, OLELNVs achieved an ABTS radical clearance rate of 82.21 %, compared to 33.17 % for OLEX. At 0.5 mg/ml, both achieved 100 % scavenging; however, OLELNVs showed higher cell viability at 116 % compared to 79 % for OLEX (Fig. 2a and d). This suggests that OLELNVs may be more effective and safer than OLEX for skin protection. At low concentrations, OLELNVs exhibited stronger free radical scavenging capacity than OLEX, likely due to their encapsulation of not only some antioxidant phenolic compounds but also certain antioxidant enzymes. In this study, the total polyphenolic content in OLELNVs was 19.77 μg/mL. Using LC/MS techniques, hydroxytyrosol was identified at 189.95 ng/mL and oleuropein at 5.84 ng/mL within the OLELNVs (Fig. S1). Additionally, commercial assay kits revealed that the OLELNVs contain 238.32 U/mL of Superoxide Dismutase (SOD), an important antioxidant enzyme that plays a critical role in the antioxidant defense system.

Transdermal absorption is a significant bottleneck in beauty and skincare that limits the effectiveness of many ingredients. As illustrated in Fig. 2f, the skin anatomy diagram, one advantage of PELNVs is their high efficacy as transdermal systems, capable of deep penetration into the stratum corneum. PELNVs, with a lipid bilayer structure like liposomes commonly used in skincare, penetrate the skin *via* the transfollicular route [21,22]. Additionally, penetration can occur through transcellular and intercellular pathways across the stratum corneum. Given the similarity between the membrane surfaces of PELNVs and mammalian cells, it is proposed that PELNVs can effectively traverse the stratum corneum *via* both pathways through lipid fusion effects [23]. In an *in vitro* study, broccoli exovesicles were demonstrated to maintain morphological integrity and exhibit a high encapsulation rate, with notably enhanced penetration through the stratum corneum. Compared to free fluorescent molecules, those encapsulated within exosomes show improved penetration and distribution within the stratum corneum and dermis [24]. In this study, Dil-labeled OLELNVs (OLELNVs-Dil) and dithranols-labeled OLEX (OLEX-DNS) were employed to assess their transdermal efficacy using Franz diffusion cells. Unlike OLEX-DNS, which primarily remains on the skin surface, OLELNVs-Dil were extensively distributed within the epidermis and reached the dermis, confirming the superior transdermal capabilities of OLELNVs (Fig. 2g and h).

Through comparative testing, it has been established that OLELNVs not only exhibit stronger antioxidant effects than OLEX but also demonstrate greater cell compatibility and superior transdermal permeability. Consequently, OLELNVs hold promise as an advanced alternative to OLEX in skin care applications.

### 3.3 Inhibition of UV-induced cellular aging by OLELNVs

**Fig. 3:**
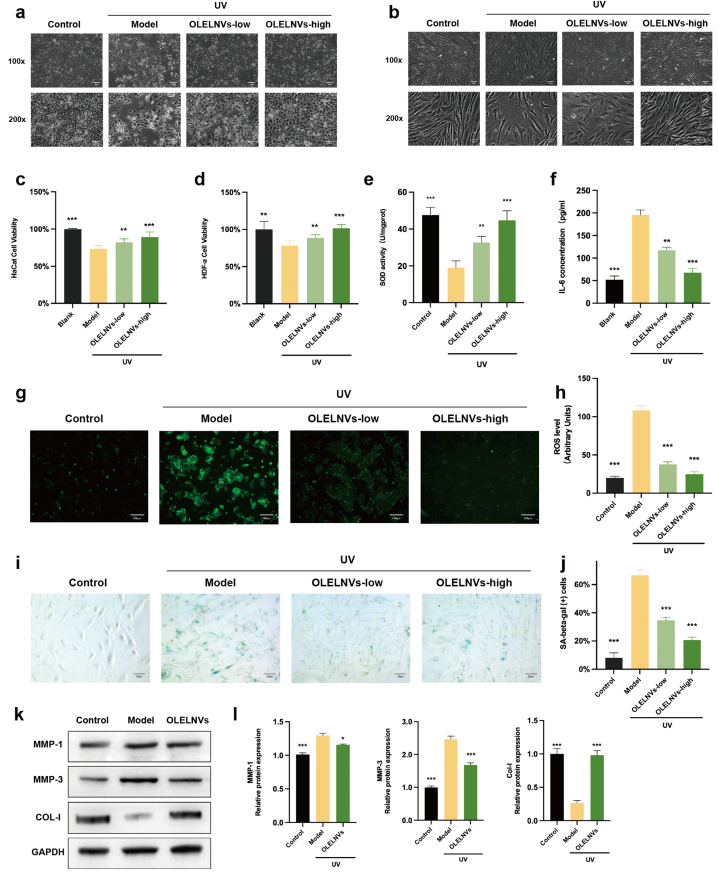
Inhibition of UV-induced cellular aging by OLELNVs. (a, b) The effect of OLELNVs on the morphology of UVB-treated HaCaT and HDF-α cells. Model: UVB-treated. Scale bar = 40 μm, 20 μm, n = 3. (c, d) The effect of OLELNVs on the proliferation of UVB-treated HaCaT and HDF-α cells by CCK8 assay, n = 3. (e) Relative SOD enzyme activity, (f) relative Il-6 levels, (g, h) relative ROS levels in UVB-treated HaCaT cells. Scale bar = 100 μm. (i, j) Relative SA-β-gal positivity rates in UVB-treated HDF-α cells. Scale bar = 20 μm, n = 3. (k, l) The effect of OLELNVs on the expression of MMP-1, MMP-3 and COL-I protein in UV-treated HDF-α cells was tested by western blot, n = 3. Data are expressed as mean ± SD (*P＜0.05, **P＜0.01, ***P＜0.001, NS: not significant）.

UV radiation induces oxidative stress, leading to inflammation and aging in line with the oxidation-inflammation theory of aging proposed by De La Fuente and Miquel (2009b) [25]. Oleuropein, the primary component of OLEX, acts as an antioxidant, protecting the skin from UVB-induced damage [26,27]. This study aimed to explore whether OLELNVs could similarly mitigate oxidative stress, inflammatory reactions, and photoaging induced by UV radiation in human epidermal and dermal cells. To this end, UV damage models were established using HaCaT and HDF-α cells. Initially, CCK8 assays were employed to assess the proliferative effects of OLELNVs on HaCaT and HDF-α cells. Post-UV induction, microscopic observations revealed slowed cell proliferation, increased nuclear volume, and the emergence of more synapses, dark spots, and fragments in both HaCaT and HDF-α cells (Fig. 3a and b). In addition, the cell viability of both cell lines was inhibited. However, subsequent treatment with OLELNVs at various concentrations alleviated this damage in a dose-dependent manner (Fig. 3c and d).

After exposure to UV radiation, skin cells produce reactive oxygen species (ROS), disrupting the balance of the cellular antioxidant system and leading to oxidative stress. ROS, and SOD activity as mentioned earlier, serve as crucial indicators for assessing oxidative stress levels [28]. Additionally, UV exposure prompts skin cells to produce significant amounts of inflammatory factors, notably interleukin-6 (IL-6), which triggers both local and systemic inflammatory responses [29]. Further investigations were conducted into the effects of OLELNVs on UV-induced ROS in HaCaT cells, as well as their impact on SOD activity and inflammatory factors. The findings revealed that OLELNVs reduce ROS and IL-6 levels in a dose-dependent manner and restore SOD activity (Fig. 3e–h).

Moreover, UV-induced senescent cells typically exhibit increased volume and express biological markers of skin aging, such as elevated activity of senescence-associated β-galactosidase (SA-β-Gal). The influence of OLELNVs on SA-β-Gal activity in HDF-α cells was examined, with a dose-dependent decrease observed following OLELNV treatment (Fig. 3i and j). Western blot analysis confirmed that OLELNVs significantly counteract the UV-induced upregulation of matrix metalloproteinases (MMP-1 and MMP-3), which are involved in collagen degradation. Consequently, OLELNVs restored the levels of type I collagen (COL-I) diminished due to UV exposure (Fig. 3k and l). These results suggest that OLELNVs may effectively mitigate UV-induced cellular damage and aging in human skin cells.

### 3.4 Potential cross-kingdom regulatory activity of OLELNVs

**Fig. 4:**
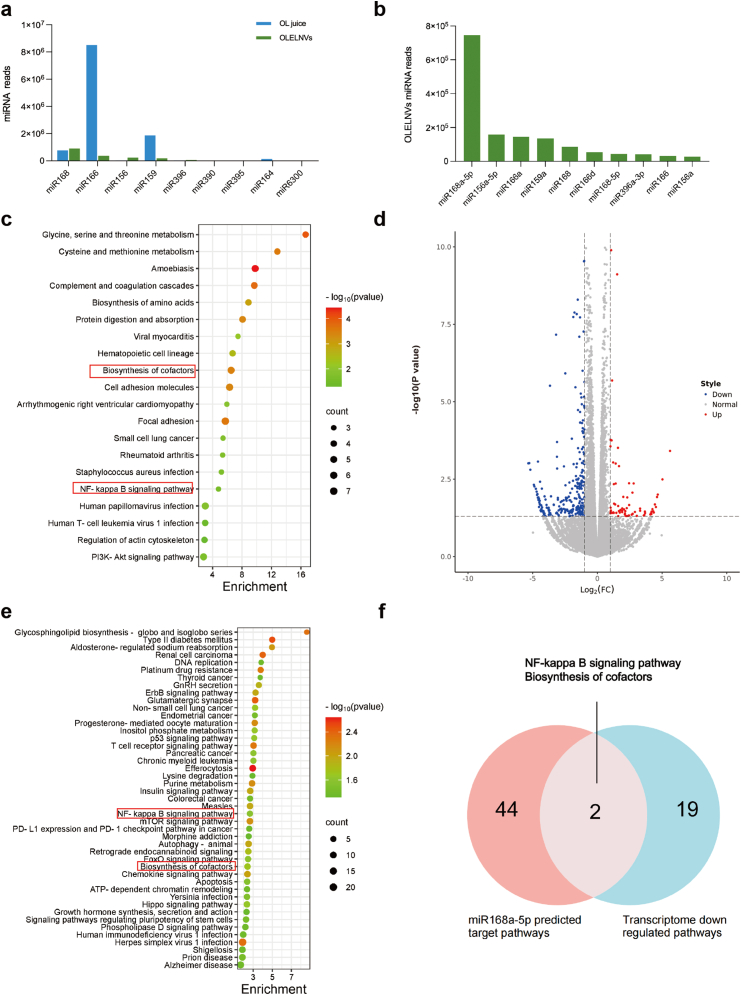
Potential cross-kingdom regulatory activity of OLELNVs. (a) Top 9 most abundant miRNA families in OLELNVs and OL juice. (b) Top 10 most abundant miRNAs of OLELNVs. (c) The KEGG analysis results of human target genes predicted by miR168a-5p in OLELNVs. (d) The differentially expressed genes in OLELNVs-treated UVB-induced HaCaT cells compared with the UVB-induced cells. (e) The KEGG analysis results of differentially expressed genes in the OLELNVs-treated group after UVB radiation. (f) The Venn plot result of both enriched signaling pathways.

Studies have shown that extracellular vesicles contain high levels of miRNAs, which are integral to the post-transcriptional regulation of gene expression and may influence various pathological and physiological processes [30]. In this study, experiments were conducted using intact OLELNVs, isolated RNA from OLELNVs, RNA-free OLELNVs (treated with RNase A to degrade RNA components), and RNase A as a control. The results demonstrated that the RNA within OLELNVs plays a significant role in protecting the viability of HaCaT cells from UVB (Fig. S2). Therefore, this study aimed to investigate the miRNA within OLELNVs. Total RNA was extracted from *O. europaea* leaf juice (OL juice) and OLELNVs and analyzed using capillary electrophoresis in the Qsep100 automated nucleic acid protein analysis system. The analysis revealed distinct peaks within the 10-10k nt range in OL juice, while OLELNVs primarily showed concentration in the 100 nt range, with a predominant peak at 20–24 nt and minimal or no presence of 18S and 28S ribosomal RNA, confirming the presence of miRNAs within OLELNVs (Fig. S4). Subsequent construction of miRNA libraries for OL juice and OLELNVs, followed by sequencing, generated a total of 13,460,897 raw reads (Fig. S5). After stringent filtering, 13,361,052 reads were classified as reliable miRNA candidates. Mapping these sequences to miRBase identified 2263 unique known miRNA sequences. In OL juice, the miR166 family is the most abundant (73.80 %), followed by the miR159 family (16.24 %), with the third most abundant being the miR168 family (6.65 %), which primarily originates from OLELNVs (Fig. 4a). Notably, within OLELNVs, the miR168 family comprised 49.01 % of all miRNAs, with miR168a-5p representing 40.51 % (Fig. 4b). Literature indicates that the miR168 family in higher plants significantly influences apoptosis, immune function, and anti-inflammatory processes in mammalian and human cells. For instance, rice MIR168a modulates cholesterol metabolism in mice by suppressing LDLRAP1 expression [31], and FvmiR168 from strawberries interacts with dendritic cells by binding to TLR3, reducing cytokine release and inhibiting T cell proliferation [32].

To further explore the potential functions of the OLELNV-derived miRNAs, bioinformatics analysis predicted the relationship between the miRNAs and their potential target genes using TargetScan and Miranda, based on seed region complementarity. This analysis predicted human target genes and signaling pathways associated with the highly enriched miR168a-5p in OLELNVs (Fig. 4c). Furthermore, transcriptomic analysis *via* Illumina sequencing technology was employed to examine the expression of differentially expressed genes between UVB-exposed and OLELNV-treated UVB-exposed groups (Fig. 4d). KEGG enrichment analysis, performed by Metascape.org, revealed significant down-regulation of the NF-κB signaling cascade, a key mediator of inflammatory responses, following OLELNV treatment (Fig. 4e). The intersection of enriched pathways confirmed the NF-κB signaling pathway as a critical overlap, suggesting a potential pharmacological mechanism by which OLELNVs mitigate inflammation and aging (Fig. 4f).

### 3.5 Enhanced stability of OLELNVs in HA/TA hydrogel

The proven efficacy of OLELNVs opens a wide range of application possibilities in the field of skin care products and topical dressings. However, the long-term preservation of natural ELNVs remains a significant challenge. The most common preservation methods for ELNVs involve freezing and freeze-drying to powder −80 °C [33]. Despite their effectiveness, these methods are not suitable for everyday product use. In solution, ELNVs are subject to irregular Brownian motion, leading to aggregation, membrane rupture, and subsequent inactivation of their contents without membrane protection [34]. Therefore, developing a method to limit the movement and aggregation of ELNVs under refrigerated or even room temperature conditions could represent a novel strategy for long-term preservation. Studies have shown that the incorporation of ELNVs into hydrogels can maintain or even enhance the stability of proteins and microRNAs within ELNVs [35]. It has been demonstrated that loading ELNVs with GelMA hydrogel restricts random movement and reduces vesicle aggregation, thereby allowing long-term storage of vesicles at 4 °C [14]. This finding inspires us to explore the potential of improving the stability of OLELNVs by encapsulating them within a hydrogel network.

**Fig. 5:**
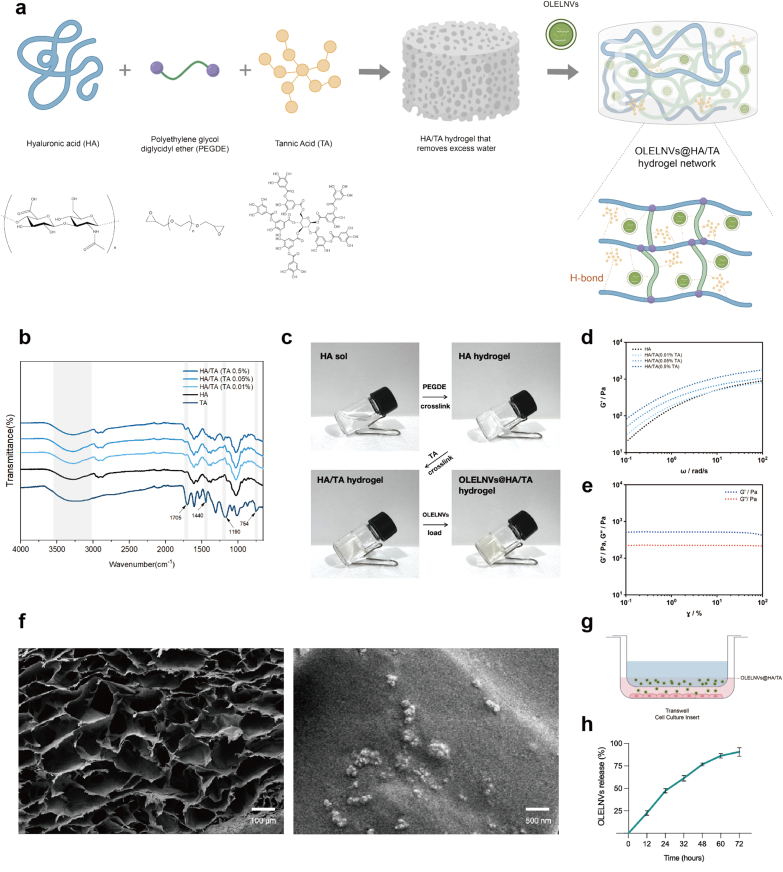
Preparation and characterization of HA/TA and OLELNVs@HA/TA hydrogels. (a) Schematic representation of the cross-linking process of hydrogels. (b) ATR-IR spectra of HA, TA and HA/TA. (c) Photograph of the sol-gel transition of HA/TA and OLELNVs@HA/TA hydrogels. (d) Rheological property of the formed HA/TA hydrogel at different TA concentrations (TA: 0 %, 0.01 %, 0.05 %, 0.5 %) in frequency sweep. (e) Rheological property of the formed OLELNVs@HA/TA (TA: 0.05 %) hydrogel in strain sweep. (f) SEM images of OLELNVs@HA/TA (TA: 0.05 %). Scale bar = 100 μm, 500 nm. (g) Schematic representation of OLELNVs@HA/TA in a Tranwell insert. (h) The cumulative release rate of OLELNVs was quantitatively measured using proteins. (mean ± SD, n = 3).

The schematic diagram illustrating the production of the hydrogel is presented in Fig. 5a. The formation of the hydrogel involves both covalent and physical cross-linking mechanisms. Initially, during the cross-linking of HA, the hydroxyl groups of HA react with the epoxy groups of PEGDE, forming covalent ether bonds. Given that TA possesses a high density of hydroxyl groups, it subsequently forms hydrogen bonds with various macromolecules within the HA hydrogel. This intermolecular interaction between TA and PEGDE enhances the cross-linking density of the hydrogel. HA, a linear, non-sulfated glycosaminoglycan, is the primary component of the extracellular matrix (ECM) and is present in almost all body tissues and fluids, offering excellent biocompatibility [36]. TA, a natural polyphenol abundantly distributed in plant tissues, protects cells from UV radiation damage caused by due to its UV absorption properties [37]. To incorporate OLELNVs into the HA/TA hydrogel, the "breathing method" was employed: OLELNVs were co-cultured with the cross-linked HA/TA hydrogel that removed excess water, resulting in the formation of the OLELNVs@HA/TA hydrogel network.

The IR spectra revealed that the stretching vibration peak of the carbonyl C=O in HA/TA appeared at 1705 cm^−1^, and the stretching vibration peaks of the aromatic C=C in TA were observed at 1190 cm^−1^ and 1440 cm^−1.^ Additionally, the peak at 754 cm^−1^ corresponded to the bending vibration of C–H on the benzene ring. The addition of TA broadened the –OH peak due to the association of hydroxyl groups from HA and TA *via* hydrogen bonding (Fig. 5b). This analysis of the infrared spectrum confirms the successful synthesis of the HA/TA hydrogel. Fig. 5c illustrates the hydrogel formation process. Rheological testing indicated that the elastic modulus (G′) of HA/TA hydrogels increased significantly with higher TA concentrations, likely due to the increased hydrogen bond cross-linking reactions between TA and cross-linked HA (Fig. 5d). The elastic modulus (G′) of the OLELNVs@HA/TA hydrogel was below 1000 Pa but exceeded the viscous modulus (G″), suggesting that the formed hydrogel was in a soft, solid-like state with high structural stability, making it suitable for skincare applications (Fig. 5e). To further characterize the internal structure and microstructure of the hydrogel loaded with OLELNVs, OLELNVs@HA/TA was observed using scanning electron microscopy (SEM). The hydrogels exhibited a three-dimensional network structure, with the distribution of OLELNVs particles within the hydrogel clearly visible (Fig. 5f).

**Fig. 6:**
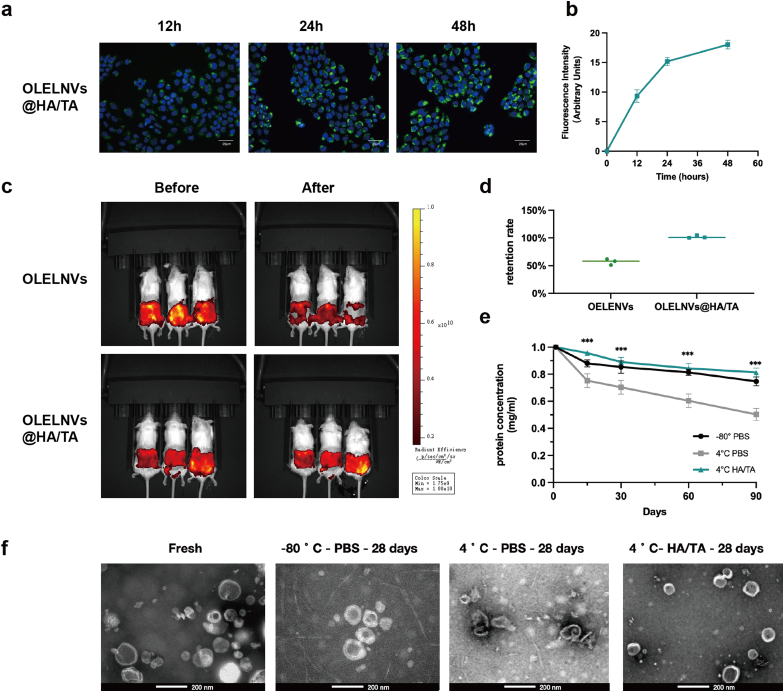
Enhanced stability of OLELNVs in HA/TA hydrogel. (a, b) PKH67‐labeled OLELNVs encapsulated in the HA/TA hydrogel remain in HaCaT cells for different periods. Scale bar = 20 μm, n = 3. OLELNVs were labeled with PKH67 (green) and HaCaT cell nuclei with DAPI (blue). (c, d) Dil-labeled OLELNVs and OLELNVs@HA/TA were applied to the dorsal skin of mice. Skin retention was imaged with IVIS before and after being sprayed with artificial sweat, n = 3. (e) Protein quantifications of OLELNVs preserved by different methods for different periods. n = 3 per group. (f) TEM images of OLELNVs preserved by different methods after 28 days. Scale bar = 200 nm. Data are expressed as mean ± SD (***P＜0.001). (For interpretation of the references to colour in this figure legend, the reader is referred to the Web version of this article.)

To verify the positive effect of HA/TA hydrogel encapsulation on OLELNVs, a cumulative release test, a skin sweat resistance test, and a storage stability test of OLELNVs were conducted. First, Transwell inserts were used to evaluate the sustained release capability of the OLELNVs@HA/TA hydrogel system (Fig. 5g). *In vitro*, OLELNVs encapsulated in HA/TA hydrogels (TA: 0.05 %) exhibited long-term stable release (Fig. 5h). Fluorescence micrographs of PKH67-labeled OLELNVs@HA/TA in HaCaT cells also showed continuous release of OLELNVs within the gel system and their uptake by skin cells, resulting in sustained delivery of their cargo molecules to the cells (Fig. 6a and b). Secondly, free ELNVs have a relatively short half-life in the skin as they are rapidly degraded by body fluids such as sweat and are exposed to external stimuli [43]. The aim is to investigate whether the encapsulation of OLELNVs with HA/TA could enhance their stability on moist skin. To this end, artificial sweat was sprayed on the skin surface of mice to evaluate changes in the half-life of OLELNVs under moist skin conditions. The IVIS images clearly show that the fluorescence of free OLELNVs was significantly weaker after continuous spraying with water, while the adhesion of OLELNVs loaded on HA/TA hydrogel to the skin remained stable, with the fluorescence signal well preserved even after continuous washing with water (Fig. 6c and d). This indicates that HA/TA hydrogels increase the stability of OLELNVs on the skin. Additionally, the stability of OLELNVs was tested under different storage conditions. TEM results showed that fresh OLELNVs had a round vesicle structure surrounded by a standard bilayer membrane. After one month, OLELNVs stored conventionally in PBS at 4 °C showed significant aggregation, disruption of the vesicle structure, and marked damage to the vesicle membrane. In contrast, OLELNVs stored in PBS at −80 °C and in HA/TA hydrogels at 4 °C exhibited relatively normal vesicle structure and size, comparable to the fresh vesicle structure (Fig. 6f). Furthermore, it has been indicated that both freezing at −80 °C and storage in HA/TA hydrogels at 4 °C effectively prevented a significant decrease in the protein concentration of OLELNVs. Over a storage period of three months, the protein concentration of OLELNVs freezing at −80 °C and loaded in HA/TA hydrogels at 4 °C decreased by about 20%–25 %. In comparison, the concentration of OLELNVs decreased more rapidly when stored at 4 °C in PBS, decreasing by 50 % after three months (Fig. 6e). Therefore, strong evidence supports the belief that the HA/TA hydrogel network can successfully encapsulate OLELNVs, create a sustainable delivery mechanism for OLELNVs, and improve their stability.

### 3.6 Synergy of HA/TA hydrogel with OLELNVs in inhibiting cellular damage and photoaging in mouse skin

To further explore the role of HA/TA hydrogel in combination with OLELNVs in protecting skin against photoaging, a UV-induced *in vitro* model using HDF-α cells and an *in vivo* mouse skin photoaging model for validation were developed.

**Fig. 7:**
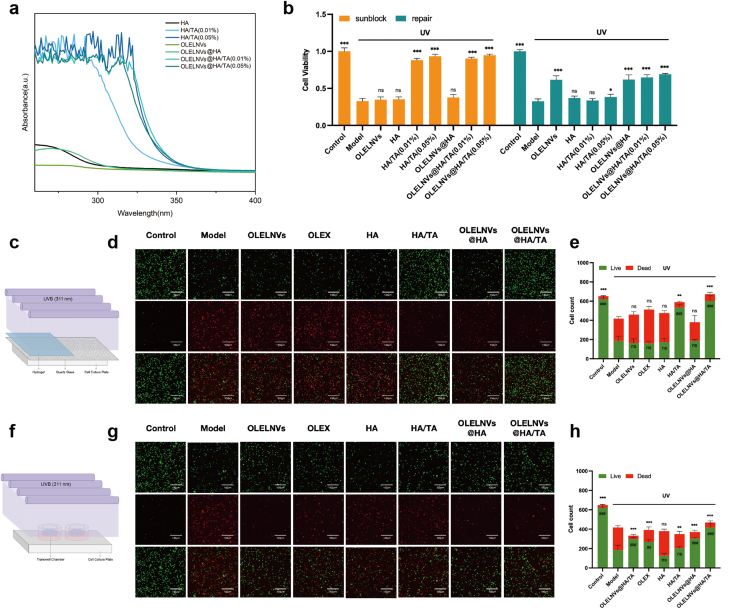
Synergy of HA/TA hydrogel with OLELNVs in inhibiting cellular damage.(a) UV–vis absorption spectra at 330 nm of HA, OLELNVs@HA, HA/TA and OLELNVs@HA/TA in different ratios. (b) The effect of OLELNVs, HA, OLELNVs@HA, HA/TA and OLELNVs@HA/TA on the proliferation of HDF-α cells in UVB block and damage-repair models by CCK8 assay, n = 3. (c) Schematic representation of the UVB block model. (d, e) Cell viability/cytotoxicity of HDF-α cells in a UVB block model was measured with live and dead staining reagents. Scale bar = 100 μm, n = 3. (f) Schematic representation of the UVB damage-repair model. (g, h) Cell viability/cytotoxicity of HDF-α cells in the UVB damage-repair model was measured with live and dead staining reagents. Scale bar = 100 μm, n = 3. Data are expressed as mean ± SD (*P＜0.05, **P＜0.01, ***P＜0.001, NS: not significant).

The UV absorbance of HA/TA hydrogels loaded with different TA concentrations, both with and without OLELNVs was measured to evaluate the UV resistance of the HA/TA hydrogel system. HA, OLELNVs and OLELNVs@HA exhibited minimal absorbance in the UVA (320∼400 nm) and UVB (280∼320 nm) wavelength ranges, indicating that HA and OLELNVs lack UV-blocking properties. Conversely, the HA/TA hydrogel demonstrated a significant UV-blocking effect, which increased with higher TA content (Fig. 7a). This effect is attributed to TA's polyphenolic structure, which acts as a sunscreen by absorbing UV radiation in the UVB range and certain areas of the UVA range [38]. Additionally, CCK8 assays and live/dead cell staining on HDF-α cells were performed. To simulate sunscreen application, a UVB blocking model was developed. Quartz plates coated with OLELNVs, HA hydrogel, HA/TA hydrogel at different TA concentrations, and OLELNVs@HA/TA hydrogel were placed on cell culture plates to block UVB radiation, followed by 48 h of cell incubation (Fig. 7c). For comparison, a UVB damage repair model was employed: after UVB irradiation of cell culture plates without quartz plate cover, the tested samples were placed in the Transwell inserts and incubated with the cells for 48 h (Fig. 7f). The results showed that in the UVB blocking model, the HA/TA and OLELNVs@HA/TA groups reduced UVB-induced cell viability damage, while the OLELNVs, HA and OLELNVs@HA groups showed no significant difference from the model group. In the UVB damage repair model, the OLELNVs and OLELNVs@HA/TA groups showed remarkable restoration of cell viability, whereas the HA and HA/TA groups did not exhibit significant repair abilities (Fig. 7b). Live/dead cell staining experiments confirmed the strong UV barrier ability of HA/TA (Fig. 7d, e and S6), the repair ability of OLELNVs after UV exposure (Fig. 7g, h and S7) and the dual UV damage resistance of OLELNVs@HA/TA. These results indicate that the combined action of TA in absorbing UV radiation and OLELNVs in repairing photodamage provides the strongest protection and repair for cells.

**Fig. 8:**
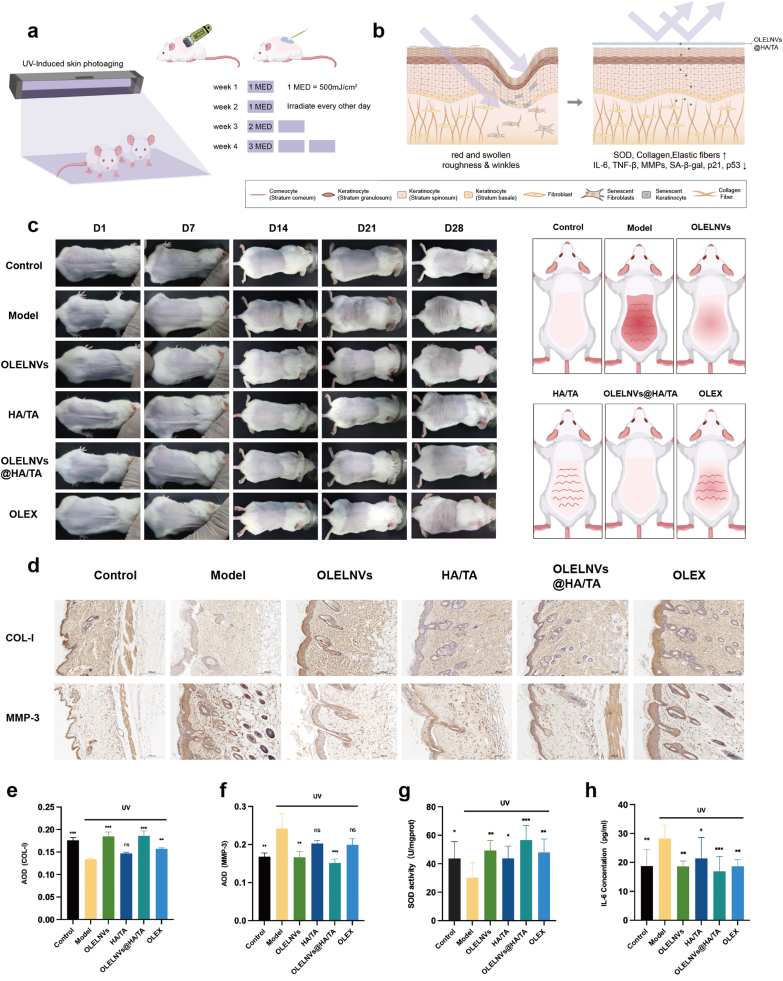
*Synergy of HA/TA hydrogel with OLELNVs in inhibiting photoaging in mouse skin*. (a) Schematic representation of the experimental setup. (b) Anatomical representation of the skin changes before and after treatment. (c) Representative photos of the dorsal skin of mice on day 1, 7, 14, 21 and 28 after the first treatment. Schematic representation of mice on day 28 after the first treatment. (d–f) Representative immunohistochemistry (IHC) images of the dorsal skin of mice after 4 weeks of treatment. Scale bar = 100 μm, n = 3. (g) Relative SOD activity and (h) relative IL-6 concentration of dorsal skin of mice after 4 weeks of treatment, n = 10. Data are expressed as mean ± SD (*P＜0.05, **P＜0.01, ***P＜0.001, NS: not significant).

Photoaging leads to a deterioration of the skin's appearance, including symptoms such as wrinkles, sagging, redness, and hyperpigmentation. UVB radiation exacerbates these conditions by inducing collagen and elastic fiber degradation, oxidative stress, and inflammation. The experimental design for the mouse skin photoaging model is shown in Fig. 8a. The treatment groups included OLELNVs, HA/TA, OLELNVs@HA/TA and OLEX, with weekly photo documentation. After 28 days, back skin samples were collected from the mice to assess the effects of the treatment groups on UVB-induced aging. The model group exhibited marked thickening, redness and deep wrinkles on the back skin, while the treatment groups showed varying degrees of improvement: the OLELNVs group had slightly reddened skin but no wrinkles, the HA/TA group had no sunburn but some wrinkles, the OLEX group had slight redness and fine wrinkles, and the OLELNVs@HA/TA group showed no obvious signs of photoaging (Fig. 8c). The absence of sunburn in the HA/TA and OLELNVs@HA/TA groups may be attributed to TA's ability to absorb ultraviolet light, providing sun protection. The different degrees of wrinkle improvement observed in the OLELNVs, OLEX, and OLELNVs@HA/TA groups may be due to the reparative effects of *O. europaea* leaves on the skin.

**Fig. 9:**
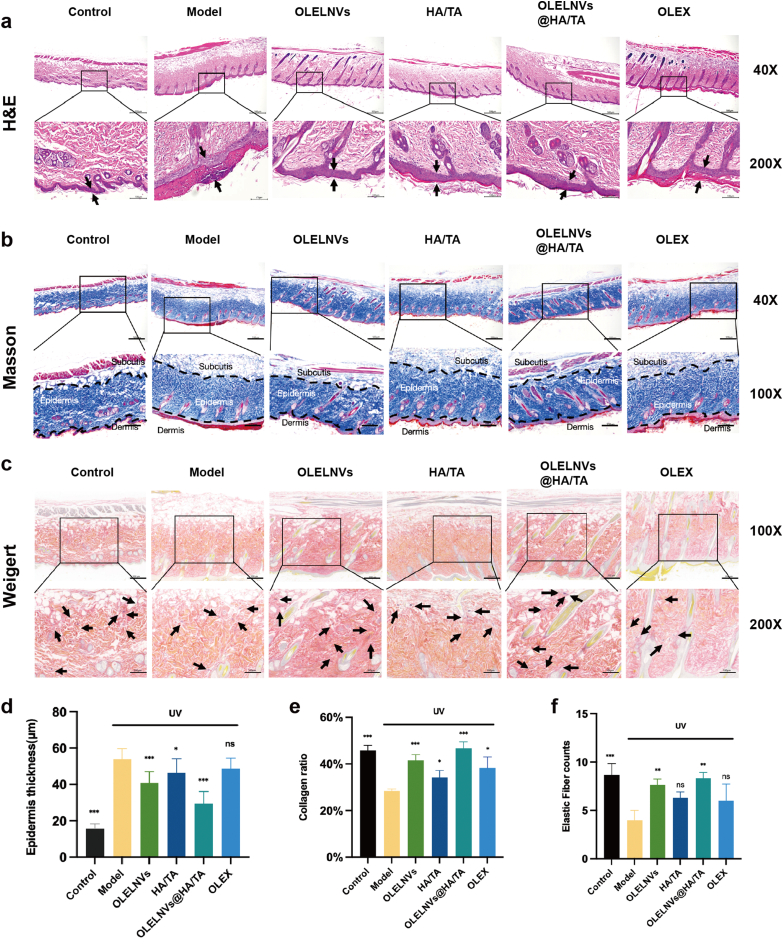
Pathological analysis of mouse skin tissue. (a) Representative images of H&E staining and (b) Masson staining of mice after 4 weeks of treatment. Scale bar in full view = 500 μm. Scale bar in magnified partial view = 100 μm. (c) Representative images of Weigert staining of mice after 4 weeks of treatment. Scale bar in full view = 200 μm. Scale bar in magnified partial view = 100 μm. (d–f) Statistical data on epidermal thickness (n = 10), collagen content (n = 4) and number of elastic fibers (n = 3) of the dorsal skin tissue of mice.

Histological analyses further elucidated the protective effects of these treatments on a cellular level. H&E staining results showed that the model group exhibited excessive keratinization of the epidermis, thickening of the spinous layer cells and infiltration of inflammatory cells, whereas the OLELNVs, HA and OLELNVs@HA/TA groups showed varying degrees of alleviation of UV-induced skin damage. The OLELNVs@HA/TA group exhibited the best performance, with an epidermal thickness of only 29.65 ± 6.47 μm compared to 53.81 ± 5.83 μm in the model group (Fig. 9a and d). Masson staining results indicated a significant loss of collagen in the dermis of the model group, with a reduction of approximately 17.33 % compared to the control group. All treatment groups were able to increase collagen in the dermis to varying degrees, with the OLELNVs@HA/TA group showing an additional increase of 0.86 % compared to the blank control group (Fig. 9b and e). Weigert staining results indicated a reduction of elastic fibers (black dots) by almost half in the model group compared to the control group. However, both the OLELNVs and OLELNVs@HA/TA groups showed significant recovery of elastic fibers to varying degrees (Fig. 9c and f). Additionally, immunohistochemistry showed a decrease in COL-I expression and an increase in MMP-3 expression in the model group. These trends were more distinctly reversed in the OLELNVs@HA/TA group (Fig. 8d–f). These findings suggest that UV radiation significantly degrades skin collagen and elastic fibers, leading to structural changes that accelerate cellular aging. The OLELNVs@HA/TA treatment effectively mitigates these changes, preserving skin structure and function.

**Fig. 10:**
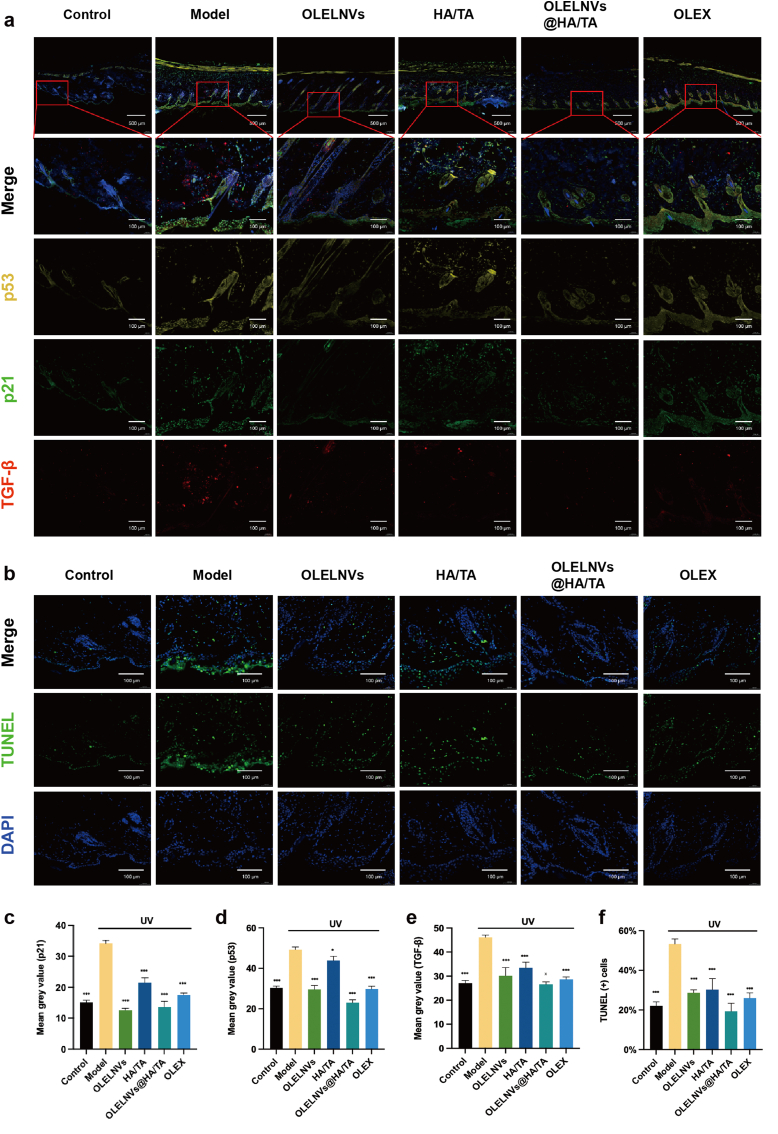
Immunofluorescence (IF) staining analysis of mouse skin tissue. (a) Representative images of IF in the dorsal skin of mice labeled for p53 (yellow), p21 (green), TGF-β (red) and DAPI (blue) after 4 weeks of treatment. Scale bar in full view = 500 μm. Scale bar in magnified partial view = 100 μm. (b) Representative images of IF in the dorsal skin of mice labeled for TUNEL (green) and DAPI (blue) after 4 weeks of treatment. Scale bar in full view = 500 μm. Scale bar in magnified partial view = 100 μm. (c–f) Statistical data on the mean gray value of p53, p21, TGF-β and TUNEL positive cell rate (n = 3) of the dorsal skin tissue of mice. Data are expressed as mean ± SD (*P＜0.05, ***P＜0.001). (For interpretation of the references to colour in this figure legend, the reader is referred to the Web version of this article.)

To further validate these findings, immunofluorescence assays were performed to assess the expression and localization of key aging indicators. TGF-β, a typical SASP factor, promotes cellular senescence by activating p53 and p21 signaling pathways. p53 is crucial in this process, activating p21, which is a CDK inhibitor that halts the cell cycle in the G1 or G2 phase by inhibiting cyclin-dependent kinases, thus preventing damaged cells from dividing further. TUNEL staining indicates apoptosis, which is closely linked to cellular senescence, where prolonged cell cycle arrest can induce apoptosis by regulating cell cycle proteins and apoptosis factors. The UV-induced model group showed the highest expression levels of p53, p21, TGF-β and TUNEL, indicating significant DNA damage and cellular apoptosis (Fig. 10a, c-e). In contrast, the OLELNVs@HA/TA group exhibited the lowest levels of these markers, suggesting that this treatment effectively reduces cellular senescence and apoptosis induced by UV radiation (Fig. 10b and f).

Lastly, to verify the antioxidant and anti-inflammatory effects of the treatment groups, biochemical assays were conducted on the skin tissue of the mice. The model group showed significantly reduced SOD activity and increased IL-6 concentration, indicating heightened oxidative stress and inflammation. All treatment groups demonstrated some degree of recovery in SOD activity, with the OLELNVs@HA/TA group showing the highest SOD activity (Fig. 8g). The IL-6 concentration in the skin tissue, except for the HA/TA group, was significantly decreased in the other treatment groups, highlighting the anti-inflammatory effect of *O. europaea* leaves (Fig. 8h).

These data collectively suggest that the OLELNVs@HA/TA hydrogel system provides comprehensive protection against UVB-induced photoaging by preserving skin structure, reducing cellular senescence, and enhancing the skin's antioxidant defenses.

## 4 Discussion

Excessive sun exposure can lead to various skin problems such as wrinkles, irregular pigmentation, rough and dry skin as well as gray-yellow and brown spots. Prolonged exposure to UV radiation accelerates the aging process and may also lead to skin cancers, including malignant melanoma and squamous cell carcinoma [39]. Therefore, there is a significant and urgent need for medications and topical dressings to improve photoaging.

OLEX has been found to repair acute and chronic skin damage caused by UV radiation, inhibiting the increase in skin thickness and the decrease in skin elasticity. It is also thought to play a role in preventing skin cancer and inhibiting the growth of surface tumors [40,41]. However, the cytotoxicity of the organic solvent extraction used in OLEX and its poor skin penetration capacity limits its clinical use. Attempts have been made to use nanoparticles to reduce cytotoxicity by adjusting the release curve, but synthetic nanoparticles often cause adverse effects such as immune system activation, inflammation, and skin allergies [42].

In response to these issues, naturally occurring PELNVs were investigated, as they have attracted interest in the cosmeceutical field due to their favorable biological properties, making them an ideal alternative to other nanocarriers in cosmeceutical products [43]. For example, PELNVs extracted from aloe vera can promote skin regeneration by activating antioxidant defense mechanisms and facilitating wound healing [44], while ginseng-derived PELNVs may exhibit anti-aging and whitening effects [45]. For the first time, ELNVs were isolated and characterized from *Olea europaea* leaves. OLELNVs range from 50 to 500 nm in size, with a peak at about 140 nm, exhibiting a spherical shape and a lipid bilayer membrane structure. OLELNVs naturally carry various bioactive molecules such as proteins, lipids and RNA, crucial for intercellular communication and molecular regulation. They are well tolerated, easily taken up by cells, and can be targeted for specific tissue absorption, showing promising potential as nanomaterials for drugs and clinical applications [46].

Furthermore, studies have shown that PELNVs contain high levels of miRNAs involved in post-transcriptional gene regulation. Plant-derived miRNAs can regulate mammalian target genes, achieving cross-kingdom regulation [47]. In this study, *in vitro* experiments confirmed that OLELNVs counteract UV-induced oxidative stress, inflammation, and aging. A miRNA library of OLELNVs revealed 2263 unique miRNA sequences, with bioinformatic analysis predicting significant enrichment in the NF-κB signaling pathway, mediating inflammation. Both *in vitro* and *in vivo* experiments demonstrated that OLELNVs have superior biocompatibility, permeability, and efficacy compared to OLEX, highlighting their potential in effective skincare.

Nevertheless, using PELNVs alone remains inadequate for addressing photoaging. Therefore, combining an active defense system to encapsulate OLELNVs offers a dual effect. Besides, natural vesicles like OLELNVs face challenges in long-term preservation and *in vivo* stability, affecting their clinical prospects. To address this, hydrogels with different concentrations of HA/TA were prepared, characterizing and testing their UV absorption capacity and encapsulation performance for OLELNVs. HA/TA stably released OLELNVs within 72 h for continuous absorption by skin cells. *In vivo* experiments simulated a humid environment, showing that OLELNVs loaded with HA/TA hydrogel had a longer-lasting effect in a sweating environment compared to their free state. Stability tests showed that OLELNVs encapsulated in HA/TA remained stable at 4 °C, suggesting that HA/TA hydrogel effectively protects OLELNVs, enhancing their stability and protecting against UV radiation.

Long-term exposure to sunlight is associated with the clinical features of skin aging. The dual strategy combats photoaging by providing both pre-sun protection and post-sun repair. This system can be applied topically to reduce the histological and clinical symptoms of skin aging. Both *in vitro* and *in vivo* experiments have shown that the HA/TA hydrogel system, with its excellent UV absorption capacity, can act as a sunscreen and synergize with OLELNVs to counteract photoaging damage. Among the treatment groups, the OLELNVs@HA/TA system performed best on all indicators. When components were used separately, the effects weakened, indicating that the superior therapeutic effect of the OLELNVs@HA/TA system results from the combined anti-aging effects of OLELNVs and HA/TA hydrogel.

## 5 Conclusion

This study developed the OLELNVs@HA/TA hydrogel system for dual protection against UV-induced skin damage. The HA/TA hydrogel not only enhances the stability of OLELNVs, providing sustained release and excellent bioavailability, but also synergizes with OLELNVs to offer dual anti-photoaging effects for both pre-sun protection and post-sun repair. In summary, the novel developed OLELNVs@HA/TA hydrogel system represents a promising therapeutic approach for treating photoaging.

## CRediT authorship contribution statement

**Zhenzhen Wang:** Writing – review & editing, Writing – original draft, Validation, Project administration, Methodology, Investigation, Formal analysis, Data curation, Conceptualization, Supervision. **Jumao Yuan:** Writing – review & editing, Software, Methodology, Data curation, Conceptualization, Formal analysis, Investigation, Writing – original draft. **Yan Xu:** Resources, Project administration, Methodology, Investigation, Writing – original draft, Writing – review & editing. **Nuo Shi:** Visualization, Resources, Funding acquisition, Conceptualization, Investigation, Supervision. **Lin Lin:** Validation, Formal analysis. **Ruirui Wang:** Methodology, Investigation, Data curation. **Rong Dai:** Validation, Supervision, Conceptualization. **Lin Xu:** Writing – review & editing, Software, Project administration, Investigation. **Ning Hao:** Writing – review & editing, Validation, Supervision, Resources. **Qianyi Li:** Writing – review & editing, Validation, Conceptualization.

## Declaration of generative AI and AI-assisted technologies in the writing process

Statement: During the preparation of this work the authors used ChatGPT4o to improve the readability and language of the final paper. After using this tool, the authors reviewed and edited the content as needed and took full responsibility for the content of the publication.

## Declaration of competing interest

The authors declare that they have no known competing financial interests or personal relationships that could have appeared to influence the work reported in this paper.

## Data Availability

Data will be made available on request.
