# Supplementary material for: Oleaeuropaea leaf exosome-like nanovesicles encapsulated in a hyaluronic acid / tannic acid hydrogel dressing with dual “defense-repair” effects for treating skin photoaging
Source: Mater Today Bio. 2024 May 31;26:101103. doi: 10.1016/j.mtbio.2024.101103 (PMC11201150; doi:10.1016/j.mtbio.2024.101103)
Supplement: Multimedia component 1 [file mmc1.docx]

**Supporting Information**

*Olea europaea* leaf exosome-like nanovesicles encapsulated in a hyaluronic acid / tannic acid hydrogel dressing with dual "defense-repair" effects for treating skin photoaging

Zhenzhen Wang ^a,f,1^, Jumao Yuan ^a,1^, Yan Xu ^e,1^, Nuo Shi ^a^, Lin Lin ^a^, Ruirui Wang ^a^, Rong Dai ^f^ , Lin Xu ^a,e,**^, Ning Hao ^g,***^, Qianyi Li ^b,c,d,2,*^

1. *Peterson's Lab, Shanghai, P.R. China*
2. *International Laboratory in Cancer, Aging and Hematology, Shanghai Jiao Tong University, School of Medicine/Ruijin Hospital/CNRS/Inserm/Côte d'Azur University, Shanghai, P.R. China*
3. *Pôle Sino-Français de Recherches en Sciences du Vivant et Génomique, Shanghai, P.R. China*
4. *Department of Emergency, Ruijin Hospital, Shanghai Jiaotong University School of Medicine , Shanghai, P.R. China*
5. *Institute of Symbolcell Biotechology, Nanjing, Jiangsu, P.R. China*
6. *Baudry Biotech. Co., Ltd, Nanjing, Jiangsu, P.R. China*
7. *College of Biotechnology and Pharmaceutical Engineering, Nanjing Tech University, Nanjing, Jiangsu, P.R. China*

*1 These authors contribute equally.*

*2 Lead Contact.*
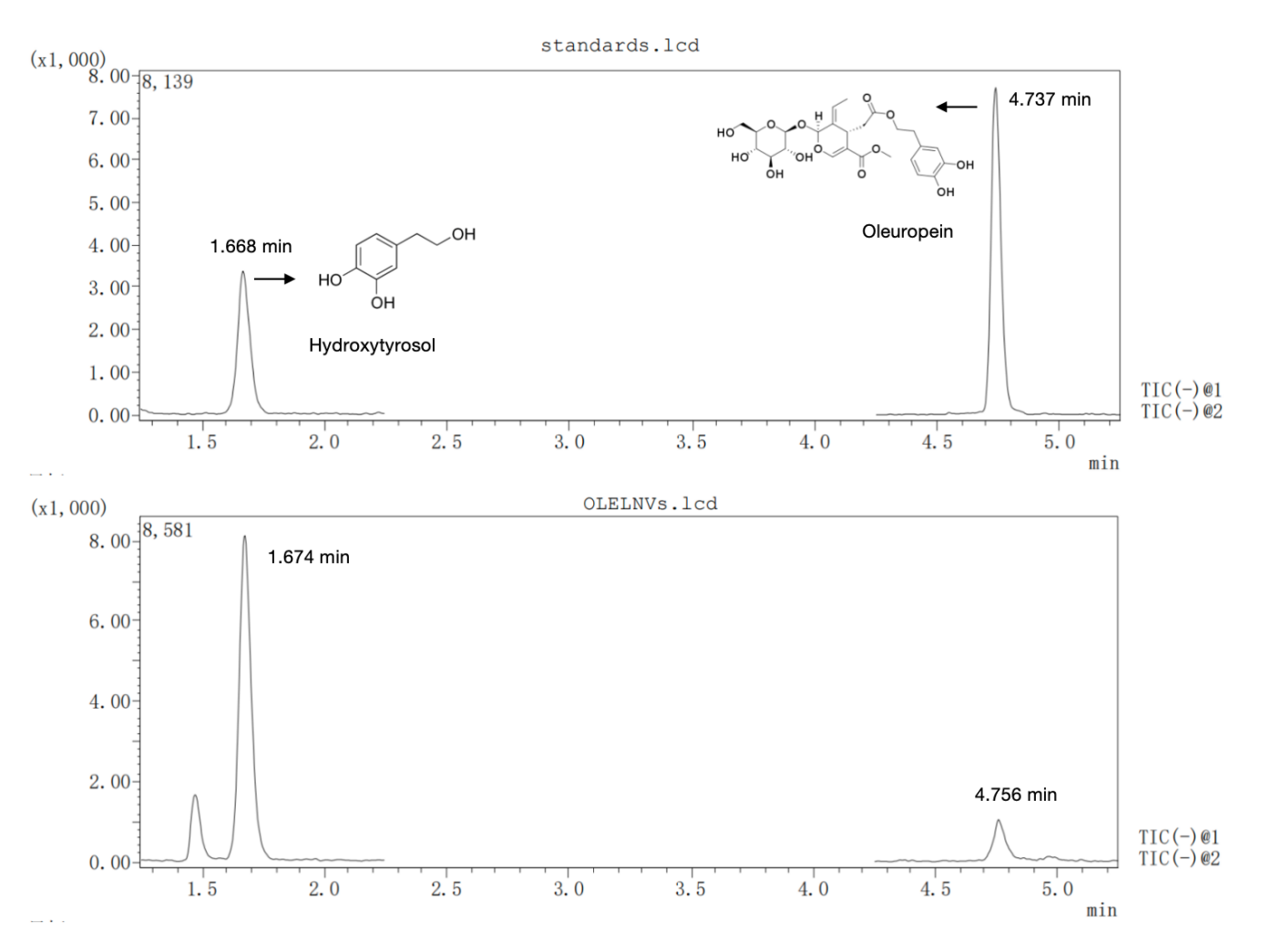


**Figure S1.** Evaluation of the contents of hydroxytyrosol and oleuropein in OLELNVs using HPLC/MS. Quantification of hydroxytyrosol and oleuropein in OLELNVs. The presence of OLELNVs were confirmed by using standards and quantified using calibration curve for each individual component.


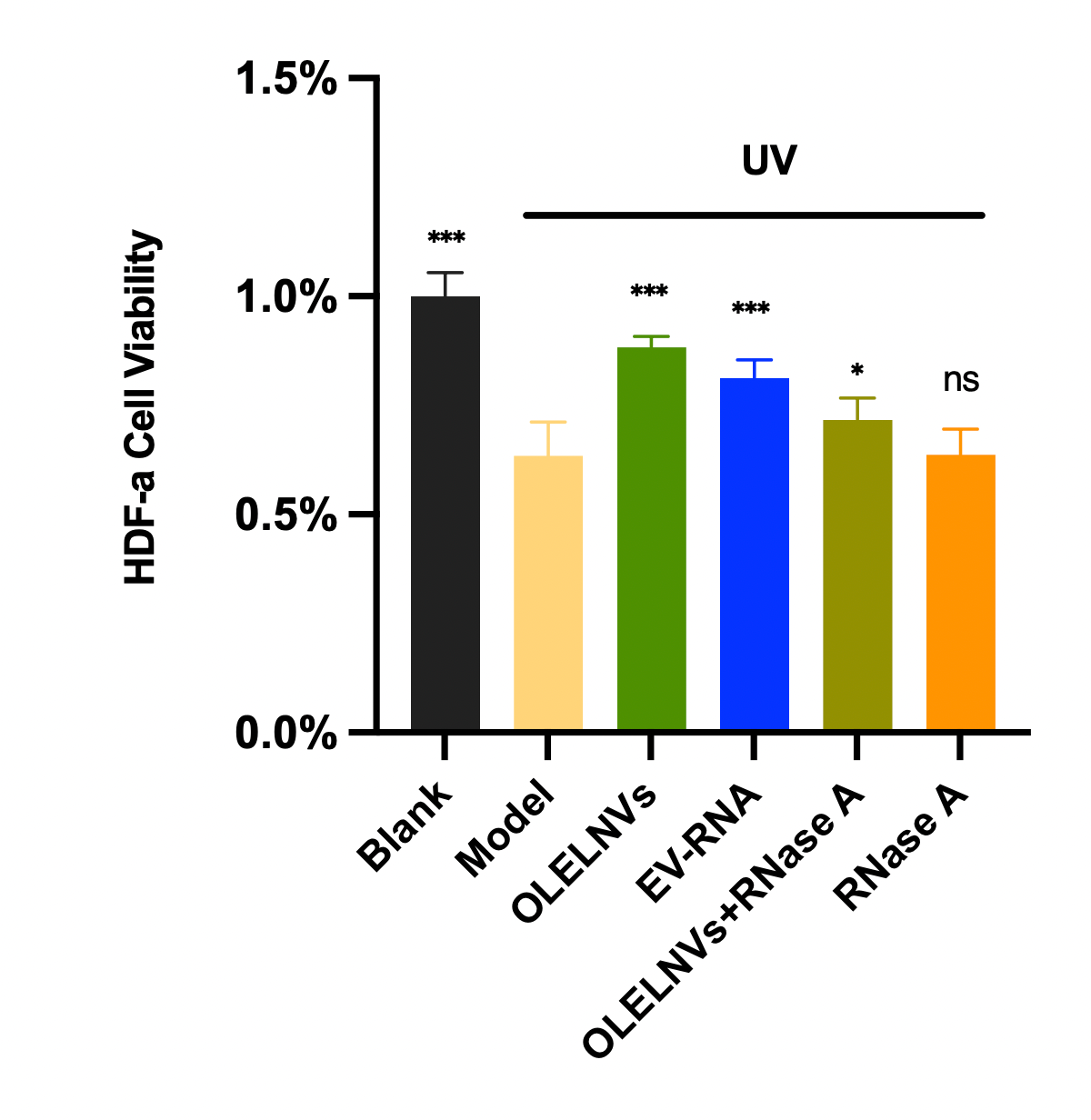


**Figure S2.** Effect of OLELNVs, OLELNVs’ RNA components (OLELNVs-RNA), OLELNVs’ RNA-free components (OLELNVs + RNase A) and RNase A as control on proliferation in UV-treated HaCaT cells by CCK8 assay, n = 6.


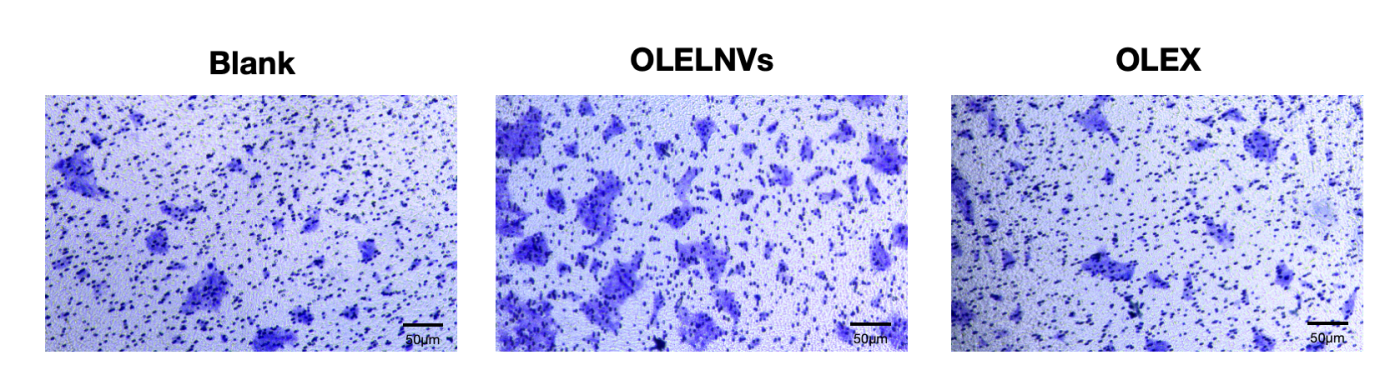


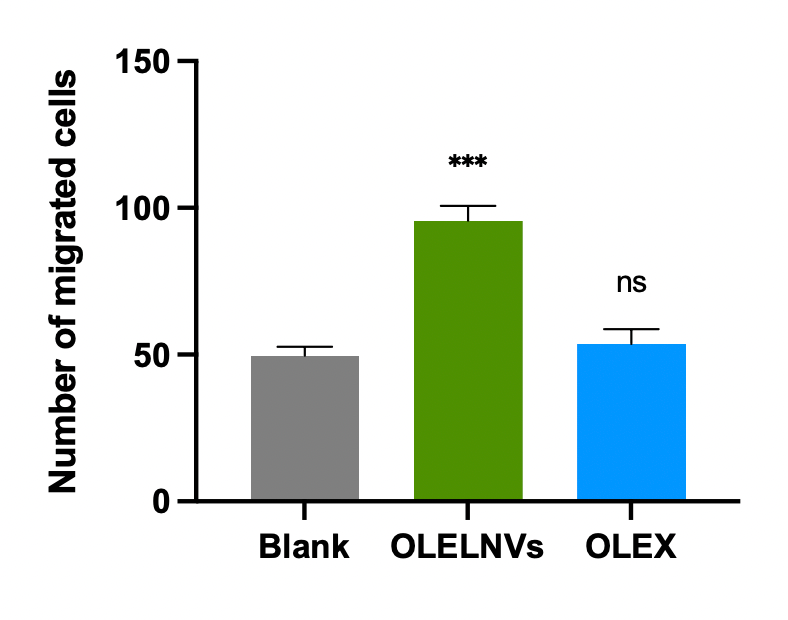


**Figure S3.** HaCaT cell migration by Transwell assay at 48 h after co-culture with OLELNVs (0.1mg/mL) or OLEX (0.1mg/mL). Scale bar = 50 μm, n = 3.


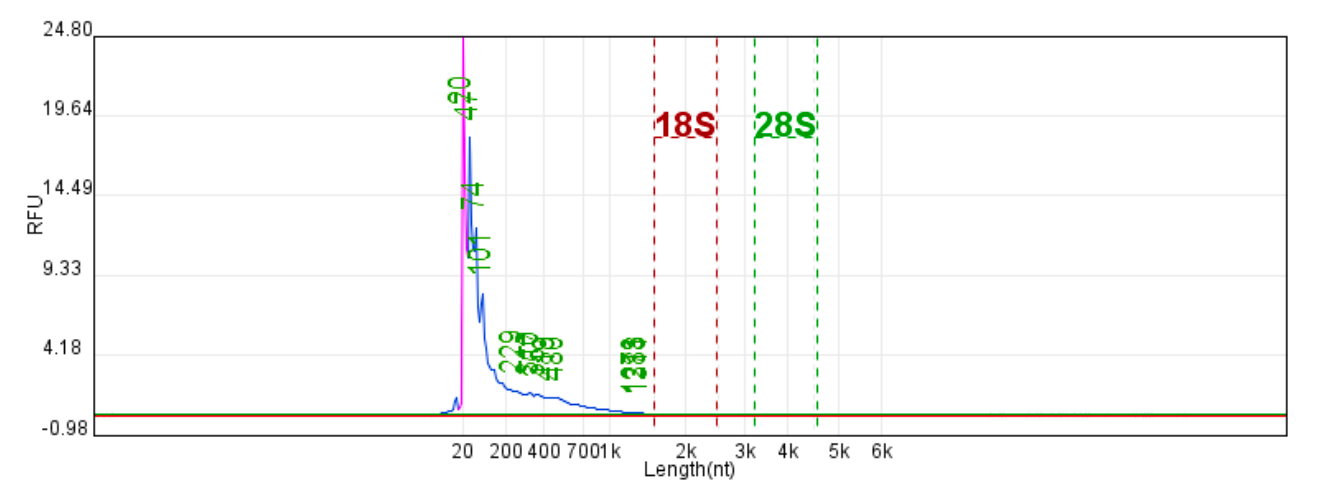


**OLELNVs**


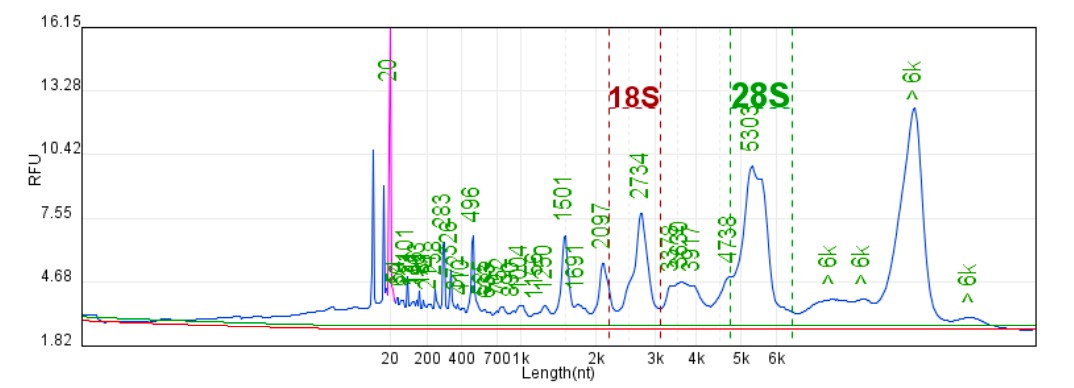


**OL juice**

**Figure S4.** RNA Qsep100 detection results of OLELNVs and OL juice.


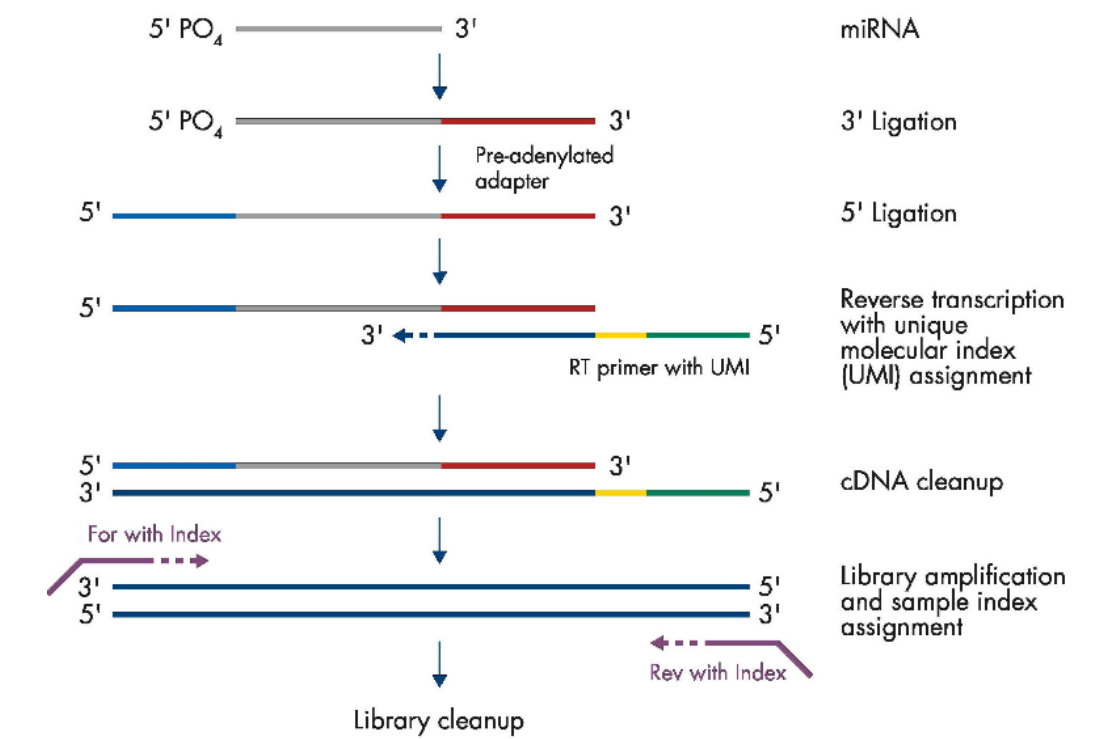
(b)

**Figure S5.** Flowchart of miRNA library preparation for OLELNVs.


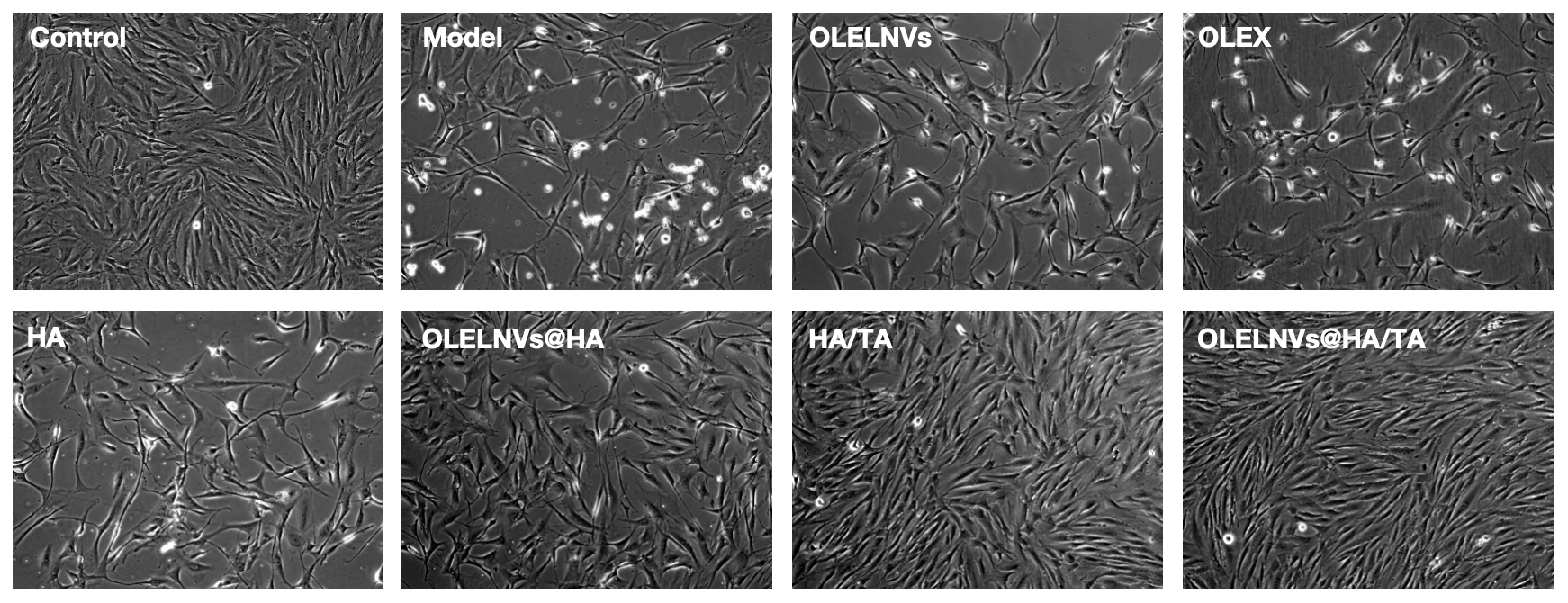


**Figure S6.** Representative images of HDF-α morphology in different groups in the UVB blocking model. Scale bar = 50 μm, n = 3.


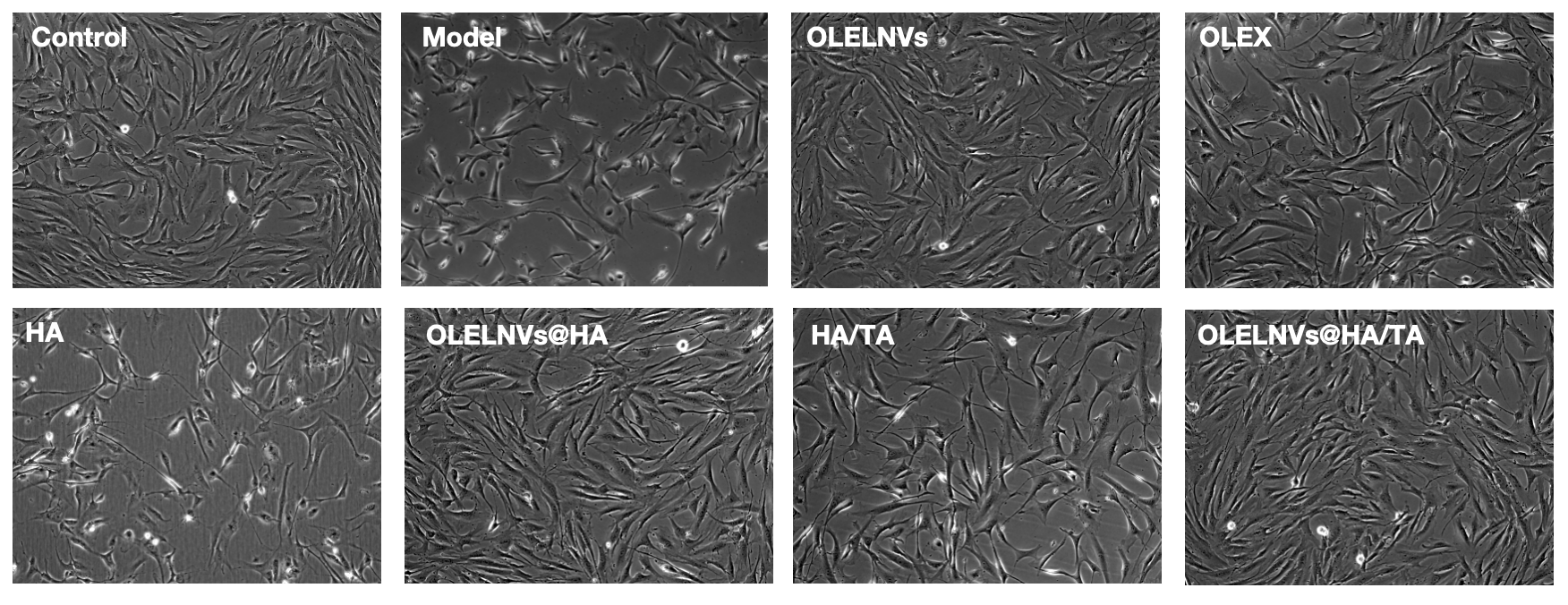


**Figure S7.** Representative images of HDF-α morphology in different groups in the UVB damage repair model. Scale bar = 50 μm, n = 3.
